# Osteoblast‐Derived Mitochondria Formulated with Cationic Liposome Guide Mesenchymal Stem Cells into Osteogenic Differentiation

**DOI:** 10.1002/advs.202412621

**Published:** 2025-01-31

**Authors:** Hye‐Ryoung Kim, Seonjeong Woo, Hui Bang Cho, Sujeong Lee, Chae Won Cho, Ji‐In Park, Seulki Youn, Gyuwon So, Sumin Kang, Sohyun Hwang, Hye Jin Kim, Keun‐Hong Park

**Affiliations:** ^1^ School of Bioconvergence CHA University 6F, CHA Biocomplex, Sampyeong‐Dong, Bundang‐gu Seongnam‐si 13488 Republic of Korea; ^2^ Department of Biomedical Science CHA University Seongnam 13488 Republic of Korea; ^3^ Department of Pathology CHA Bundang Medical Center CHA University School of Medicine Seongnam 13497 Republic of Korea

**Keywords:** delivery, liposome, mitochondria, mitochondrial transfer, MSCs, osteogenic differentiation

## Abstract

While mitochondria are known to be essential for intracellular energy production and overall function, emerging evidence highlights their role in influencing cell behavior through mitochondrial transfer. This phenomenon provides a potential basis for the development of treatment strategies for tissue damage and degeneration. This study aims to evaluate whether mitochondria isolated from osteoblasts can promote osteogenic differentiation in mesenchymal stem cells (MSCs). Mitochondria from MSCs, which primarily utilize glycolysis, are compared with those from MG63 cells, which depend on oxidative phosphorylation. Mitochondria from both cell types are then encapsulated in cationic liposomes and transferred to MSCs, and their impact on differentiation is assessed. Mitochondria delivery from MG63 cells to MSCs grown in both two‐ and three‐dimensional cultures results in increased expression of osteogenic markers, including Runt‐related transcription factor 2, Osterix, and Osteopontin, and upregulation of genes involved in Bone morphogenetic protein 2 signaling and calcium import. This is accompanied by increased calcium influx and regulated by the Wnt/β‐catenin signaling pathway. Transplantation of spheroids containing MSCs with MG63‐derived mitochondria in bone defect animal models improves bone regeneration. The results suggest that delivery of MG63‐derived mitochondria effectively guides MSCs toward osteogenesis, paving the way for the development of mitochondria‐transplantation therapies.

## Graphical abstract

This study demonstrates that the transfer of mitochondria from osteoblasts to mesenchymal stem cells (MSCs) enhances osteogenic differentiation. MSCs receiving osteoblast‐derived mitochondria exhibit increased expression of key osteogenic markers (Runt‐related transcription factor 2, RUNX2; Osterix, OSX; Osteopontin, *OPN*) and activate the BMP2 (Bone morphogenetic protein 2)‐Wnt (Wingless‐related integration site)/β‐catenin‐calcium import axis. These findings suggest that mitochondrial transfer can alter cell fate, aligning MSCs more closely with the characteristics of donor osteoblasts.

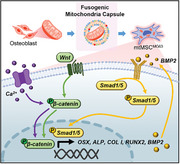

## 1 Introduction

Mitochondrial transfer is an emerging and innovative biomedical strategy with the potential to enhance cellular function and thus address various disease pathologies to alleviate chronic disease and improve overall health outcomes.^[^ ^1^ ^]^ Techniques for mitochondrial transfer include mitochondrial gene delivery, pharmacologically induced mitochondrial biogenesis, and direct mitochondrial transfer between cells or individuals.^[^ ^2^ ^]^ These strategies open the prospect of treating conditions characterized by mitochondrial dysfunction or impaired cellular energy production.

Among these approaches, mitotherapy—a method of inducing autologous healing by injecting mitochondria isolated from healthy donors into damaged cells and tissues—has been noted for its potential.^[^ ^3^ ^]^ For example, in neurodegenerative diseases, mitochondrial transfer has shown remarkable potential for enhancing cell survival and function.^[^ ^4^ ^]^ Similarly, in muscle diseases, mitochondrial therapy has significantly improved patient outcomes by restoring or protecting mitochondrial function.^[^ ^5^ ^]^

Furthermore, emerging evidence suggests that mitochondrial transfer can determine cell fate. For instance, when osteoblast mitochondria are transferred to progenitor cells, they can stimulate differentiation into mature osteoblasts.^[^ ^6^ ^]^ Furthermore, there are cases where the donor and recipient cells originate from different sources, and mitochondria from the donor cell remodel the recipient cell's metabolic activity to induce the transfer of a property from the donor to the recipient.^[^ ^2^, ^7^ ^]^ For example, the transfer of macrophage mitochondria to MSCs has resulted in the remodeling of MSC metabolic processes,^[^ ^8^ ^]^ and the transfer of MSC mitochondria to T cells induces their differentiation into regulatory T cells.^[^ ^8^ ^]^

These findings raise the possibility that the transfer of mitochondria from donor to recipient cells could ultimately determine the fate of recipient cells through mitochondrial remodeling. Herein, we set out to test this hypothesis by testing whether the transfer of mitochondria promotes osteogenic differentiation in MSCs. Specifically, we asked whether the transfer of mitochondria from osteoblasts to MSCs promotes osteoblast‐like mitochondrial metabolism in the MSCs and whether the recipient MSCs differentiate into osteogenic lineages and, if so, by what mechanism. We found that the introduction of osteoblast mitochondria into MSCs resulted in the upregulation of a number of pathways specifically related to osteoblast mitochondria metabolism and osteoblast differentiation, suggesting that osteoblast mitochondrial transfer can promote osteoblast differentiation in MSCs. These findings highlight the potential of mitochondria for the regulation of cell fate and metabolism and pave the way for an era of advanced or precision mitotherapy with various clinical applications.

## 2 Results and Discussion

### 2.1 Mitochondria from MSCs and MG63 Cells Exhibit Distinct Properties

**Figure 1:**
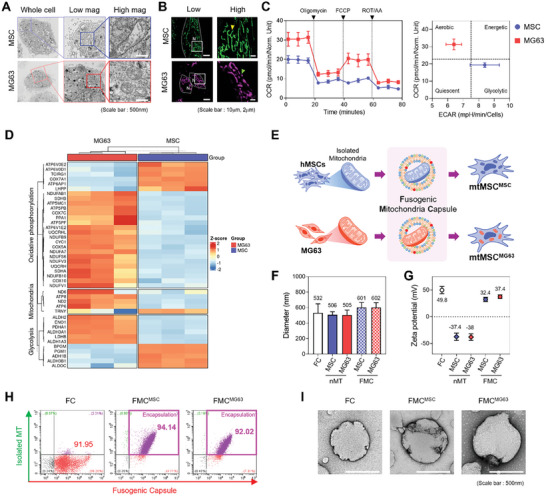
Comparative analysis of mitochondria in MG63 and MSC cells and formulation of mitochondria with cationic liposomes. A) Transmission electron microscopy and B) confocal laser scanning microscopy images comparing the mitochondrial morphological differences between MSCs and MG63 cells.  Scale bar = 500 nm, 10 µm, and 2 µm. C) Seahorse assay showing differences in cell respiration between MSC and MG63 cells (*n* = 3). D) Heatmap showing the expression levels of 42 DEGs related to glycolysis, oxidative phosphorylation, and mitochondrial genes between MG63 (*n* = 3, red) and MSC (*n* = 3, blue) samples. DEGs were grouped accordingly. DEGs were defined by a *p* value <0.05 and |log2FC| > 1. *p* values were calculated with the Wald test. The color of gene expression indicates *z*‐score of each gene. A red color indicates increased expression, while a blue color indicates decreased expression. E) A simple schematic diagram showing the experimental groups. F) Size and G) surface charge analysis of liposomes before and after mitochondrial loading (*n* = 5). H) Flow cytometry analysis confirming the encapsulation of mitochondria within fusogenic liposomes. I) TEM images showing the incorporation of MSC and MG63 mitochondria into liposomes. Scale bar = 500 nm. All data are presented as average ± SD.

To validate our approach, we first characterized the mitochondria from both donor (MG63) and recipient (MSC) cells to confirm the presence of distinct and measurable differences. Cell transmission electron microscopy (TEM) analysis revealed clear morphological differences between the two types of mitochondria (**Figure** **1**A), demonstrating that while MSC mitochondria were elongated, MG63 mitochondria were significantly more spherical. This morphological difference was further confirmed using confocal laser scanning microscopy (CLSM) analysis of mitochondria that had been stained with the mitochondria probe Mitotracker (Figure 1B). Next, we examined the mitochondrial activity of the cells using the Seahorse assay to measure the mitochondrial oxygen consumption rate (OCR) (Figure 1C). Using this analysis, mitochondria from MG63 cells exhibited a higher OCR and greater aerobic metabolism than those from MSCs, suggesting that MG63 cells have higher mitochondrial activity than MSCs. These results are consistent with previous findings that undifferentiated MSCs generate energy primarily through glycolysis rather than oxidative phosphorylation (OXPHOS).^[^ ^9^ ^]^ Furthermore, the increased mitochondrial OCR and aerobic metabolism observed in MG63 cells suggests that mitochondria may promote osteogenic differentiation in these cells.

Aerobic metabolism, which primarily uses oxygen for energy production, is a core function of mitochondria, and OCR values can represent a measure of OXPHOS activity. Therefore, these data indicate that mitochondria from MG63 cells have higher OXPHOS activity than those from MSCs, which is an active energy metabolism that produces adenosine triphosphate. These characteristics provide some evidence that replacing mitochondria in MSCs may promote osteogenic differentiation.

Next, we conducted transcriptome analysis to investigate mitochondrial features in MG63 and MSC cells. Analysis of the resultant Volcano plots demonstrated a number of differences in the expression of differentially expressed genes (DEGs) between MSCs and MG63 cells (Figure S1, Supporting Information). To more precisely identify relevant differences between the two cell types, gene ontology (GO) analysis was performed on the DEGs that reached significance using the following criteria: OXPHOS, mitochondrial deoxyribonucleic acid (mtDNA), glycolysis, mitochondrial fusion, and fission (Figures 1D and S1, Supporting Information). These analyses demonstrated that DEGs related to mtDNA and OXPHOS were upregulated in MG63 cells compared with MSCs, while DEGs related to glycolysis and mitochondrial fusion/fission showed significant variation between the two cell types.

These findings provide insights into the regulatory mechanisms that enhance the metabolic activity and differentiation potential of MG63‐derived mitochondria. Therefore, the delivery of MG63 mitochondria to recipient cells is anticipated to confer them with specific functional improvements.

### 2.2 Mitochondria Formulated with Cationic Liposomes Can Be Delivered to MSCs via Fusion with the Cell Membrane

To test our hypothesis that the transfer of mitochondria to MSCs would determine their cell fate, we sought to develop an approach for the stable and effective delivery of mitochondria extracted from MSCs and MG63 cells into MSCs. To address this challenge, we encapsulated MG63‐derived mitochondria within fusogenic liposomes, which have been developed in our lab and already shown to be highly effective^[^ ^10^ ^]^ before transferring them into MSCs, thereby preserving the functionality of the transplanted mitochondria (Figure 1E).

We indirectly confirmed mitochondria encapsulation by measuring the size and zeta potential (ZP) changes of each naked mitochondria (nMT), fusogenic capsule (FC), and fusogenic mito‐capsule (FMC) using dynamic light scattering (Figure 1F,G). This approach demonstrated that FMCs were ≈100 nm larger than FCs, indicating successful mitochondria encapsulation. When the ZP was measured, nMTs showed a negative charge of ≈−38 mV, while FCs had a charge of +50 mV. The FMCs showed a positive charge of ≈+35 mV, which is consistent with the encapsulation of the negatively charged mitochondria within positively charged liposomes. Flow cytometry was then used to confirm the successful encapsulation of mitochondria within the fusogenic liposomes. In this analysis, mitochondria from MSCs and MG63 cells, as well as fusogenic liposomes, were measured using violet side scatter filters, which allow the detection and resolution of nano‐sized particles (Figure S2, Supporting Information and Figure 1H). Unstained mitochondria (MTs) and non‐fluorescence fusogenic liposomes were used to define the negative zones, while stained MTs and fluorescent FCs were used to define the gates for MTs, FCs, and FMCs (Figure S2, Supporting Information). Flow cytometric analysis of FMC^MSC^ and FMC^MG63^ revealed successful encapsulation of 94% and 92% of mitochondria, respectively, confirming efficient mitochondrial loading into the liposomes. TEM imaging further confirmed these results, allowing the visualization of liposome‐encapsulated mitochondria (Figure 1I).

Analysis of the cytotoxicity and capacity for transfer to MSCs of mitochondria encapsulated in liposomes demonstrates that delivery at a concentration of 4 µg for 0.5 h was not cytotoxic and resulted in mitochondrial transfer at approximately double the efficiency of that for nMTs (Figure S3, Supporting Information). Thus, these conditions were used for mitochondrial transfer in all further experiments unless stated otherwise.

**Figure 2:**
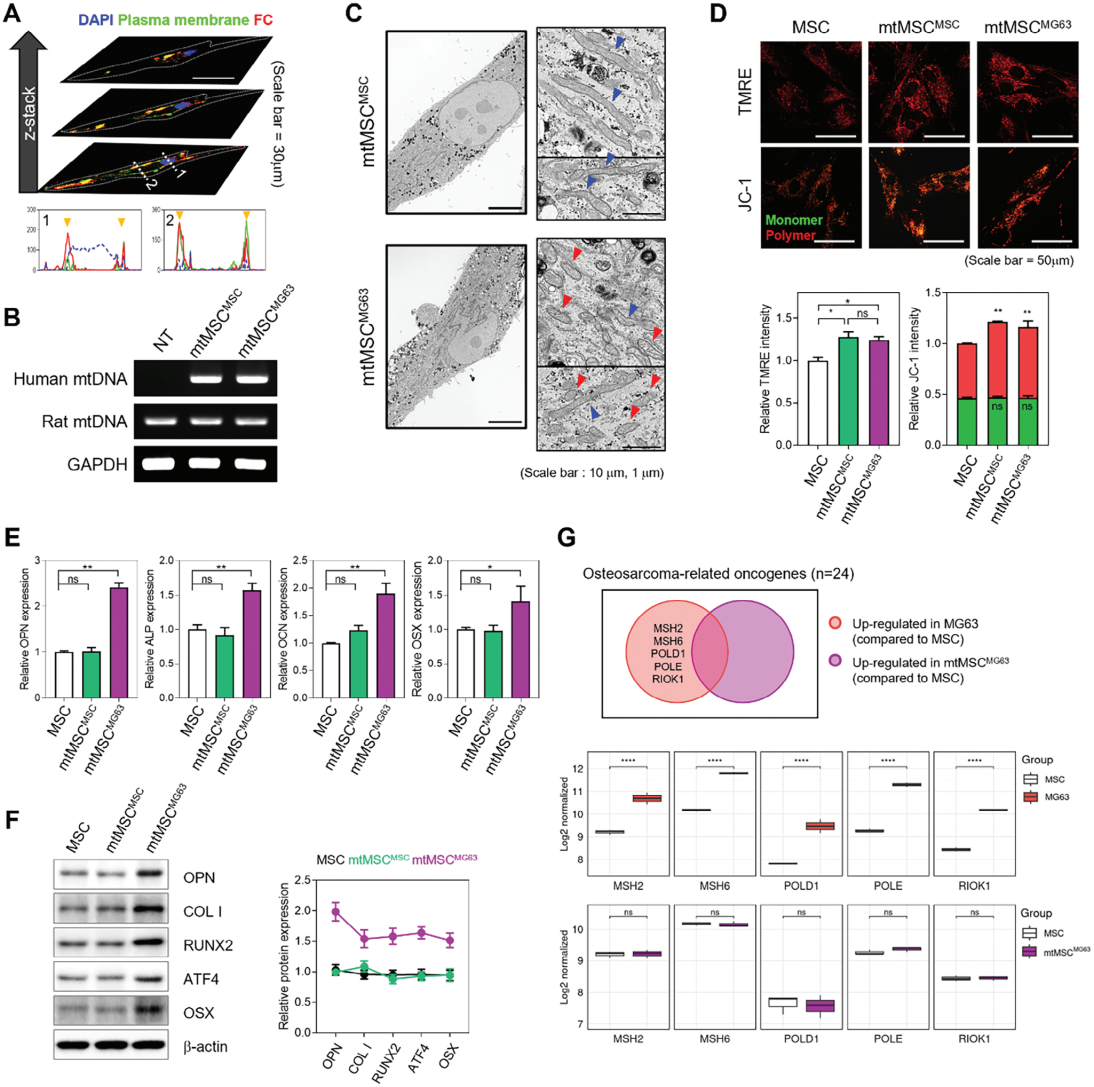
Evaluation of mitochondrial delivery and mitochondrial osteogenic differentiation effects in MSCs. A) Fusion of cationic liposomes and cell membranes observed via confocal laser scanning microscopy and intensity profiling. Scale bar = 30 µm. B) mtDNA copy number analysis to determine the extent of delivery of liposome‐encapsulated mitochondria to MSCs. C) TEM images showing mitochondrial morphological changes in mtMSC^MSC^ and mtMSC^MG63^. Scale bar = 10 and 1 µm. D) Changes in mitochondrial membrane potential were observed using TMRE and JC‐1 staining after mitochondrial transfer (*n* = 5). Scale bar = 50 µm. E) qRT‐PCR and F) western blotting analysis showing expression levels of osteogenesis‐related genes in mtMSC^MSC^ and mtMSC^MG63^ (*n* = 3). Data are presented as average ± SD (D–F); Statistical analysis was performed using unpaired Student *t*‐test; *p < 0.05, **p < 0.01, ***p < 0.001, and ****p < 0.0001. G) Venn diagram of five upregulated DEGs among twenty‐four oncogenes associated with osteosarcoma, between MG63 (*n* = 3) and MSC (*n* = 3), and mtMSC^MG63^ (*n* = 3) and MSC (*n* = 2). Boxplots showing the log_2_‐scaled normalized expression of five oncogenes in MG63 and mtMSC^MG63^ compared to MSC, with error bars represent the 95% confidence interval. DEGs were classified based on a *p* value <0.05 and |log2FC| > 1. *p* values were calculated with the Wald test. *p < 0.05, **p < 0.01, ***p < 0.001, and ****p < 0.0001.

The purpose of enclosing the mitochondria in the FC was to allow the delivery of MT to cells via fusion with the cell membrane. CLSM and subsequent quantitative analysis of cells whose nucleus and cell membrane stained with 4′,6‐diamidino‐2‐phenylindole and fluorescein isothiocyanate, respectively, and treated with FC derived from the fluorescently labeled lipid Liss Rhod PE revealed good colocalization between FC and the cell membrane (**Figure** **2**A). These results suggest that FC can efficiently deliver MT by fusing with the cell membrane.

Next, rat‐derived cell lines were treated with equal amounts of either MSC‐derived mitochondria (mtMSC^MSC^) or MG63‐derived mitochondria (mtMSC^MG63^), and mtDNA expression was confirmed using polymerase chain reaction (PCR) (Figure 2B). The results demonstrated no significant difference in the expression of the different mtDNAs between recipient cells, indicating uniform delivery of mitochondria‐encapsulated liposomes to recipient cells.

Further analysis of the mitochondria following delivery into MSCs using TEM imaging demonstrated that clear morphological differences between mitochondria derived from MSCs and those derived from MG63 cells could still be observed, enabling their identification within recipient cells post‐delivery (Figure 2C). Hence, in mtMSC^MSC^ cells. Only mitochondria with the elongated morphology characteristic of MSCs were observed, while in mtMSC^MG63^, both the elongated mitochondria of MSCs and the short and round mitochondria of MG63 cells were observed. Taken together these data confirm that we can successfully and safely transfer mitochondria to recipient cells.

We hypothesized that the delivery of osteoblast‐derived mitochondria into stem cells would significantly impact their function, differentiation, and growth, leading to changes in energy metabolism, mitochondrial activity, and regulation of cellular signaling pathways, thereby affecting cell differentiation. We further hypothesized that delivering mitochondria to stem cells may confer them with characteristics similar to those of the donor cells. Having developed and characterized a technique for mitochondrial transfer, we, therefore, set out to characterize the changes induced by the delivery of osteoblast‐derived mitochondria to MSCs.

### 2.3 MG63‐Derived Mitochondria Induced Changes in MSC Metabolism and Protein Expression

To evaluate the changes in liposome‐loaded mitochondria after their delivery into MSCs, tetramethyl rhodamine ethyl ester (TMRE) and JC‐1 assays were employed, both of which are widely used as a measure of mitochondrial membrane potential (MMP). TMRE fluorescence intensity increases in proportion to increased membrane potential, and JC‐1 fluorescence is predominantly green at low MMP and red at high MMP (Figures 2D and S4, Supporting Information). CLSM and measurements of fluorescence intensity using a plate reader revealed that mtMSC^MSC^ and mtMSC^MG63^ exhibited ≈30% higher TMRE intensity than MSC cells, indicating that mitochondrial delivery resulted in increased MMP. Furthermore, JC‐1 fluorescence was predominantly red in mtMSC^MSC^ and mtMSC^MG63^ cells, indicating that transplantation of mitochondria resulted in a more healthy and active state. Taken together, these data suggest that this approach results in the successful transfer of functional mitochondria and that mitochondrial transfer increases MMP.

Next, we investigated the effect of the delivered MG63 mitochondria on cellular respiration of mtMSC^MG63^ cells using the Seahorse assay (Figure S5, Supporting Information). MSC mitochondria are typically inactive, but after receiving MG63‐derived mitochondria, mtMSC^MG63^ cells shifted from glycolytic to aerobic metabolism and exhibited changes in the extracellular acidification rate consistent with this shift. These changes were not observed in cells treated with FC alone, indicating that these changes are dependent on the introduction of mitochondria. Taken together, these data suggest that the introduction of MG63 mitochondria into MSCs switches their cellular respiration processes to those characteristic of MG63 cells.

Furthermore, when we evaluated the effects of osteogenic differentiation markers using quantitative real‐time reverse‐transcription PCR (qRT‐PCR) in the group treated with liposomes alone under the same conditions as MT (Figure S6, Supporting Information), we found that liposomes did not have a statistically significant effect on bone formation markers. These results confirmed that liposomes alone do not influence MSCs.

To further investigate the impact of transferring mitochondria from bone to stem cells, we examined the expression of various proteins related to osteogenic differentiation. Analysis of gene expression using RT‐qPCR of osteogenic genes, including osteopontin (OPN), alkaline phosphatase (ALP), osteocalcin, and osterix (OSX), demonstrated a clear upregulation in expression patterns of these genes in mtMSC^MG63^cells relative to mtMSC^MSC^ or MSCs (Figure 2E). Furthermore, these changes in gene expression patterns translated into increased protein expression in mtMSC^MG63^ cells (Figure 2F). These results indicate that the introduction of osteocyte‐derived mitochondria into stem cells induces changes in cellular behavior and potentially enhances their differentiation capacity.

Given that the mitochondrial donor cell line was derived from osteosarcoma, we also assessed the expression of oncogenic driver genes in mtMSC^MG63^. Therefore, the expression of functional genes that maintain the osteosarcoma genome (*MSH2, MSH6, POLD1, POLE*, and *RIOK*1) was compared between MSCs and MG63 cells (Figure 2G).^[^ ^11^ ^]^ As expected, the expression of these genes was much higher in MG63 cells than in MSCs but was not higher in mtMSC^MG63^ than in MSCs. Therefore, mitochondria transplantation from MG63 cells is unlikely to increase the risk of oncogenicity in MSCs.

### 2.4 MG63‐Derived Mitochondria Promote Osteogenesis via the Bone Morphogenetic Protein 2 (BMP2)‐Wnt/β‐Catenin‐Calcium Import Axis

**Figure 3:**
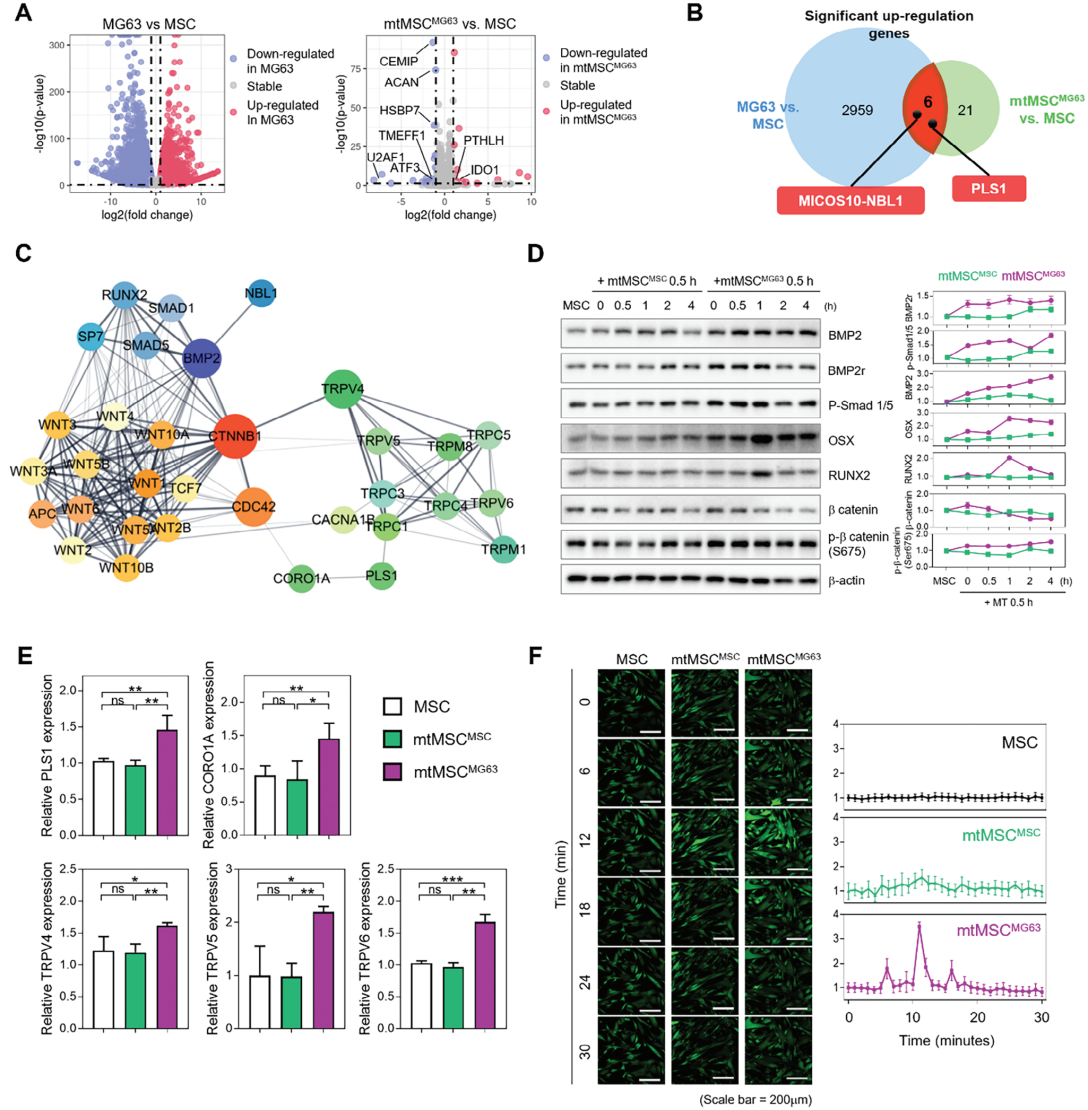
Osteogenic signaling pathways and gene expression changes induced by MG63‐derived mitochondria in MSCs. A) Volcano plots showing gene expression differences between MG63 (*n* = 3) and MSC (*n* = 3), and mtMSC^MG63^ (*n* = 3) and MSC (*n* = 2). DEGs were defined by a *p* value < 0.05 and |log2FC| >  1. *p* values were calculated using the Wald test. DEGs related to osteogenesis for both upregulated and downregulated genes were labeled in mtMSC^MG63^ compared to MSC. B) Venn diagram showing equally upregulated DEGs in the comparison in (A). C) Gene network analysis showing the BMP2‐Wnt/b‐catenin‐calcium import axis. D) BMP2 signaling and β‐catenin markers over time after delivery of each mitochondrion (*n* = 3). E) qRT‐PCR analysis comparing the expression levels of key markers of calcium import (*n* = 3). F) Confocal laser scanning microscopy image analysis of calcium influx that occurs during mitochondrial delivery (*n* = 3). Scale bar = 200 µm All data are presented as average ± SD; Statistical analysis was performed using unpaired Student *t*‐test; *p < 0.05, **p < 0.01, ***p < 0.001, and ****p < 0.0001).

We confirmed that mitochondrial transfer from MG63 promotes osteogenesis, but we wanted to understand the underlying mechanisms. To do this, we obtained transcriptomic data and performed differential expression analysis (DE analysis) to compare MSCs, mtMSC^MG63^, and MG63. We established two major comparison groups to identify statistically significant DEGs. In the MG63 versus MSC comparison, 2965 upregulated and 3573 downregulated genes were detected. In the MSC versus mtMSC^MG63^ comparison, 27 upregulated and 41 downregulated genes were identified (**Figure** **3**A). We observed that osteogenesis‐related genes such as *PTHLT*,^[^ ^12^ ^]^ *IDO1*,^[^ ^13^ ^]^ *CEMIP*,^[^ ^14^ ^]^ *ACAN*,^[^ ^15^ ^]^ *HSBP7*,^[^ ^16^ ^]^ *TMEFF1*,^[^ ^17^ ^]^ *U2AF1*,^[^ ^18^ ^]^ and *ATF3* ^[^ ^19^ ^]^ were present in the MSC versus mtMSC^MG63^ comparison and indicated them on the Volcano plot. Additionally, we investigated the genetic information of the upregulated genes in each comparison. The 2959 genes upregulated in MG63 versus MSCs were categorized into GO terms using DE analysis, showing genes related to cell‐wide metabolisms like protein binding and cell cycle (Figure S1B, Supporting Information). The 21 genes upregulated in mtMSC^MG63^ versus MSCs were primarily associated with extracellular matrix structure, enzyme activity, receptor interaction, and structural components.

Next, we believed that identifying the commonly upregulated genes in both comparison groups could provide clues to the mechanism by which MG63 mitochondria exert their effects. In other words, we analyzed the six commonly upregulated genes in both MG63 and mtMSC^MG63^, hypothesizing that there might be key factors among them (Figure 3B). Using the GeneCards database, we excluded two non‐coding ribonucleic acids (RNAs; LOC124902006 and RAD21‐AS1) and two genes (CRLF2 and CTAGE4) unrelated to osteogenesis. These genes were classified under the categories of cytokine binding and endoplasmic reticulum membrane GO terms. Ultimately, the two genes that remained were mitochondrial contact site and cristae organizing system subunit 10‐neuroblastoma suppressor of tumorigenicity 1 (NBL1), which is involved in BMP binding, and plastin‐1 (PLS1), which binds calcium ions. We identified these genes as key factors and confirmed their correlation with osteogenesis. Analysis of the protein–protein interaction (PPI) sub‐network using the STRING database revealed that mitochondrial contact site and cristae organizing system subunit 10*‐NBL1* and *PLS1*, along with their first neighbor nodes, are involved in BMP2‐Suppressor of Mothers Against Decapentaplegic (SMAD) signaling (blue cluster) and calcium import (green cluster), respectively. Furthermore, we found that a Wnt/β‐catenin signaling cluster exists between these two clusters (Figure 3C).

Key genes involved in BMP‐2 signaling include SMAD, which is a pivotal mediator in the BMP‐2 pathway,^[^ ^20^ ^]^ and Runt‐related transcription factor 2 (RUNX2), which is crucial for osteocyte differentiation. RUNX2, in conjunction with OSX, plays a vital role in the differentiation and maturation of osteocytes.^[^ ^21^ ^]^ In the Wnt/β‐catenin signaling pathway, β‐catenin regulates gene expression related to osteoblast differentiation and osteogenesis, and its role may vary depending on the phosphorylation state of β‐catenin.^[^ ^22^ ^]^ The combined action of BMP‐2 and WNT/β‐catenin signaling pathways contributes to the differentiation of MSCs into mature osteoblasts and the subsequent formation of mineralized bone tissue.^[^ ^23^ ^]^

We first investigated the differences in expression levels of the core factors corresponding to the NBL1‐BMP2 axis and the Wnt/β‐catenin cluster at the messenger RNA (mRNA) and protein levels and compared their expression between mtMSC^MSC^ and mtMSC^MG63^. The mRNA expression level of NBL1 was significantly increased in mtMSC^MG63^ (Figure S7, Supporting Information), and the expression levels of its associated proteins BMP2, BMP2r, phospho‐SMAD1/5, RUNX2, and OSX were all elevated in mtMSC^MG63^ cells (Figure 3D). Furthermore, the expression levels of WNT2 and WNT3 were significantly higher in mtMSC^MG63^ cells than in mtMSC^MSC^ cells and MSCs, and the expression of phosphorylated β‐catenin (Ser675) was also higher in mtMSC^MG63^ cells (Figure S7, Supporting Information and Figure 3D). Phosphorylated β‐catenin (Ser675) translocates to the nucleus and acts as a transcription factor to promote the transcription of osteogenic factors such as RUNX2 and OSX.^[^ ^24^ ^]^ Therefore, we hypothesize that MG63‐derived mitochondria act as an activator that stimulates p‐β‐catenin Ser675 and promotes MSC osteogenesis by stimulating the BMP‐2 signaling pathway and enhancing related factors.

The Wnt inhibitor IWP2 suppresses Wnt production, leading to a reduction in β‐catenin expression levels.^[^ ^25^ ^]^ Based on this reference, we treated cells with IWP2 for 48 h, followed by treatment with MG63 mitochondria for 1 h. We then evaluated the levels of phospho‐β‐catenin S675, along with the expression of the BMP2 signaling pathway. In the IWP2‐treated group, we observed a decrease in phospho‐β‐catenin S675, along with a reduction in BMP2, p‐SMAD1/5, and RUNX2, which are components of the BMP2 signaling pathway. This suggests that phospho‐β‐catenin S675, acting as a transcription factor, stimulates the BMP2 signaling pathway and promotes the transcription and expression of related factors. In contrast, the group treated with MG63 mitochondria showed an increase in the expression levels of these factors. However, in the group co‐treated with MG63 mitochondria and IWP2, no increase was observed (Figure S8A, Supporting Information).

Next, we conducted an experiment to activate p‐β‐catenin S675 using forskolin (FSK) as an activator.^[^ ^26^ ^]^ FSK was applied for 30 min, followed by treatment with MG63 mitochondria, and the results were evaluated. In the FSK‐treated group, the levels of p‐β‐catenin S675 increased, and we observed a concurrent increase in the expression of BMP2‐related markers. This indicates a positive correlation between the increase in p‐β‐catenin S675 and the promotion of osteogenesis. Similarly, the MG63 mitochondria‐treated group exhibited the same effect as FSK, suggesting that MG63 mitochondria function similarly to FSK. This trend was also observed in the group where FSK and MG63 mitochondria were applied simultaneously. We repeated the experiment at a different time point, 24 h later, and observed similar results (Figure S8B,C, Supporting Information). These results demonstrate that MG63 mitochondria play a functional role in activating phospho‐β‐catenin S675.

BMP2 is a representative growth factor that promotes bone formation; thus, given that the expression of the receptor (BMP2r) also increased, levels of secreted BMP2 were also analyzed. Consistent with the western blotting results, the amount of BMP2 secreted from mtMSC^MG63^ cells was significantly higher than that secreted from mtMSC^MSC^ cells and increased in a time‐dependent manner (Figure S9, Supporting Information).

Next, we investigated the PLS1‐calcium import axis (green cluster). Our DE analysis showed that it was closely correlated with the Wnt/β‐catenin signaling pathway. Our initial analyses demonstrated that the expression of β‐catenin and its first neighbor, *CDC42*, was increased at the mRNA level (Figure S7, Supporting Information). Therefore, we next analyzed the expression levels of the linked genes *CORO1A* and *PLS1*. The expression levels of both *CORO1A* and *PLS1* were higher in mtMSC^MG63^ cells than in MSCs and mtMSC^MSC^ cells. Furthermore, the expression levels of transient receptor potential vanilloid (TRPV)4, another first neighbor of β‐catenin, and its neighbors TRPV5 and 6 were higher in mtMSC^MG63^ cells than in the other groups (Figure 3E).

Given that TRPV4 is a calcium‐permeable ion channel and our analyses demonstrated significant differences in the expression of genes related to calcium import, we next investigated the influx of calcium ions into cells. To explore this, mitochondria were delivered to cells that had been stained with Flou‐4, and the change in intracellular calcium ion influx that occurred upon mitochondria delivery was observed in real time (Figures 3F and S10, Supporting Information). After mitochondria delivery, Flou‐4 intensity increased in mtMSC^MSC^ and mtMSC^MG63^ cells compared with that in MSCs, and the increase was particularly noticeable in mtMSC^MG63^ cells, suggesting that calcium influx occurs when mitochondria are delivered to cells, particularly MG63‐derived mitochondria. The increase in calcium influx in mtMSC^MG63^ cells likely increases the expression of TRPVs.

Taken together, these data confirm that MG63‐derived mitochondria but not MSC‐derived mitochondria regulate the complex action of the BMP2 pathway centered on Wnt/β‐catenin signaling and calcium influx, ultimately leading to the differentiation of MSCs into osteogenic lineages.

### 2.5 MG63‐Derived Mitochondria Promote Osteogenic Transcriptome Expression in the Three‐Dimensional (3D) Spheroid State

**Figure 4:**
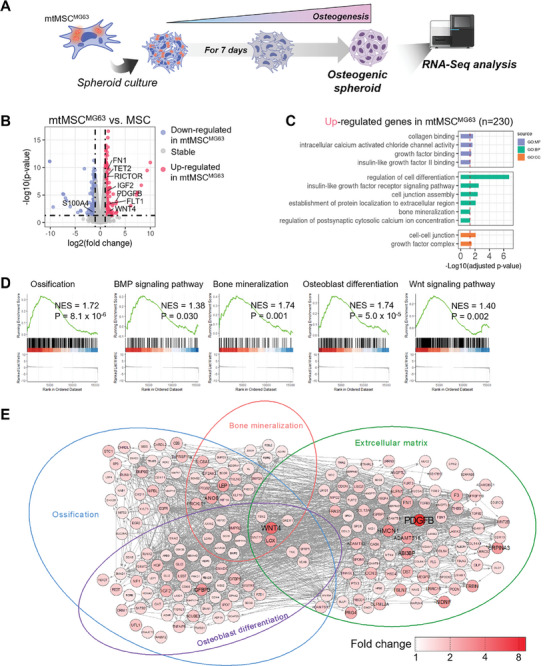
DE analysis and evaluation in 3D MSC spheroids after MG63‐derived mitochondria transfer. A) Schematic diagram of the analysis conducted following osteogenic differentiation of mtMSC^MG63^. B) Volcano plot showing DEGs between mtMSC^MG63^ (*n* = 2) and MSC (*n* = 2) spheroids 7 days after mitochondrial delivery. DEGs were defined by a *p* value < 0.05 and |Log_2_FC| >  1. *p* values were calculated using the Wald test. C) GO functional analysis of upregulated DEGs in mtMSC^MG63^ spheroid. *p* values were calculated using Fisher's exact test and adjusted with the FDR method. The colors represent molecular function (MF, light navy), biological process (BP, green), and cellular component (CC, orange) of GO. D) Gene set enrichment analysis (GSEA) of genes related to the osteogenic differentiation pathway in mtMSC^MG63^ spheroids. The normalized enrichment score (NES) represents the statistical significance of a gene set's overrepresentation within a ranked list of genes in mtMSC^MG63^ spheroids compared to MSC spheroids. The heatmap indicates the z‐scores of individual genes, where red represents increased expression and blue represents decreased expression. *p*‐values were calculated using permutation tests and adjusted with the FDR method. E) A gene network of the mtMSC^MG63^ spheroid was associated with osteogenic differentiation. The color of the node represents the fold change of mtMSC^MG63^ spheroid compared to MSC spheroid.

So far, we confirmed that mtMSC^MG63^ cells undergo significant changes during the osteogenic differentiation process, while MSCs and mtMSCs do not. Therefore, we used MSCs as a control group in all further experiments involving mtMSC^MG63^ cells. To compare the degree of osteogenic differentiation more directly, we investigated the expression of osteogenic transcriptome genes in cells grown as 3D spheroids (**Figure** **4**A). The spheroid model has limitations in mimicking complex interactions within the body owing to its low heterogeneity and singularity. However, as a tissue composed of a single cell type, it is convenient for assessing the efficacy on specific cells. Additionally, in 3D cultures, the interactions between cells function more effectively, making it a model closer to in vivo conditions compared to than conventional 2D cell cultures.

DE analysis demonstrated that the osteogenesis genes *FN1*,^[^ ^27^ ^]^ *TET2*,^[^ ^28^ ^]^ *RICTOR*,^[^ ^29^ ^]^ *IGF2*,^[^ ^30^ ^]^ *PDGFB*,^[^ ^31^ ^]^ *FLT1*,^[^ ^32^ ^]^ *WNT4*,^[^ ^33^ ^]^ and *S100A4* ^[^ ^34^ ^]^ were up or downregulated in mtMSC^MG63^ cells compared with that in MSCs (Figure 4B). When we investigated for enriched GO terms among the 230 upregulated DEGs, pathways associated with osteogenic differentiation, including collagen binding, regulation of cell differentiation, insulin‐like growth factor receptor signaling, and bone mineralization, were enriched in mtMSC^MG63^ cells (Figure 4C). Gene set enrichment analysis also revealed that ossification, BMP signaling, bone mineralization, osteoblast differentiation, and Wnt signaling pathways were significantly upregulated in mtMSC^MG63^ cells (Figure 4D), along with mitochondrial adenosine triphosphate synthesis coupled electron transport, mitochondrial protein (containing complex), and nitric oxide metabolic processes (Figure S11, Supporting Information). These results support the hypothesis that mitochondria from MG63 cells can induce osteogenic differentiation genes in MSCs.

We then mapped the osteogenic gene network based on the upregulated DEGs in mtMSC^MG63^ cells (Figure 4E). This network was organized into four key categories: ossification, bone mineralization, osteoblast differentiation, and extracellular matrix (ECM). This analysis revealed that DEGs within these categories were interconnected, indicating a coordinated network. These findings suggest that MG63‐derived mitochondria impact osteogenic differentiation through a complex gene network involving ossification, bone mineralization, and ECM formation.

Next, we repeated the analysis of the expression of functional genes associated with the osteosarcoma genome in 3D spheroids derived from mtMSC^MG63^ cells (Figure S12, Supporting Information). In 2D cultured cells, no significant differences in the expression of osteosarcoma genes were observed between mtMSC^MG63^ and MSC spheroids. This suggests that there is no delayed upregulation of osteosarcoma‐associated genes following the transfer of MG63‐derived mitochondria.

MSCs differentiate into three major lineages: osteogenic, chondrogenic, and adipogenic. To investigate the impact of mitochondrial transfer on alternative differentiation pathways, we assessed the extent of chondrogenic and adipogenic differentiation at 24 h and seven days after the transfer of mitochondria from MG63 cells and MSCs (Figure S13, Supporting Information). At 24 h, mtMSC^MSC^ exhibited no statistically significant differences in chondrogenic (COLII, SOX9, and COMP) or adipogenic markers (C/EBPα, SREBP1, and adiponectin) compared with control MSCs. In contrast, mtMSC^MG63^ displayed significantly reduced expression of all these markers relative to MSCs and mtMSC^MSC^. These findings, which differ from the trends observed for osteogenic differentiation markers, suggest that mitochondrial transfer selectively facilitates osteogenesis.

Additionally, in 7‐day cultures of mtMSC^MG63^, the expression levels of previously reduced markers returned to levels comparable to those of MSCs. Based on these results, we conclude that osteoblast‐derived mitochondria strongly drive MSCs toward osteogenic differentiation from the early stages, with minimal influence on alternative differentiation pathways.

### 2.6 MG63‐Derived Mitochondria Promote ECM Expression and Guide MSCs toward the Osteogenic Lineage

Next, we investigated the changes occurring in the later stages of MSC osteogenic differentiation. To thoroughly evaluate the effects of MG63‐derived mitochondria, we compared the experimental group with two control groups. The first control group consisted of MSCs alone, while the second control group comprised MSCs treated with liposomes without mitochondria (MSC^Lipo^), allowing us to distinguish the specific impact of MG63‐derived mitochondria on osteogenic differentiation from any changes arising from the liposomes used for mitochondrial delivery.

**Figure 5:**
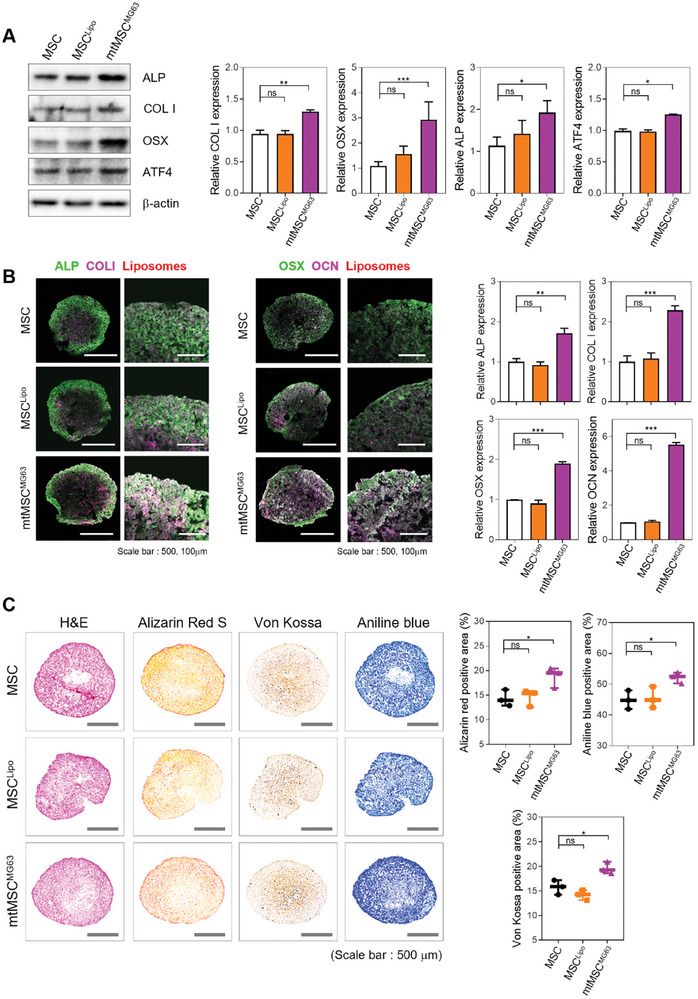
Osteogenic protein expression in 3D MSC spheroids after MG63‐derived mitochondria transfer. A) Western blot analysis of osteogenic differentiation protein expression in spheroids of MSC, mtMSC^Lipo^, and mtMSC^MG63^ (*n* = 3). B) Immunostaining analysis of osteogenic differentiation protein expression in spheroid sections of MSC, mtMSC^Lipo^, and mtMSC^MG63^ (*n* = 3). Scale bar = 500 and 100 µm. C) Histological staining of spheroid sections of MSC, mtMSC^Lipo^, and mtMSC^MG63^ (*n* = 3). Scale bar = 500 µm. All data are presented as average ± SD. Statistical analysis was performed using an unpaired Student *t*‐test; *p < 0.05, **p < 0.01, ***p < 0.001, and ****p < 0.0001.

After mitochondria delivery, the recipient MSCs were cultured in 3D spheroids for seven days, and the degree of differentiation into bone cells was evaluated using western blot analysis. The differentiation degree was assessed by analyzing the expression of key differentiation‐related proteins, including ALP, type I collagen (COL I), activating transcription factor 4, and OSX, as described in earlier experiments. In all cases, the expression of key osteogenic differentiation proteins was significantly higher in mtMSCMG63 than in MSC and MSC^Lipo^ (**Figure** **5**A). This finding confirmed that by aligning the metabolic profiles of donor and recipient stem cells through mitochondrial transfer, the osteogenic differentiation potential of stem cells can be enhanced.

To further substantiate these findings, we examined the expression of the above‐mentioned differentiation‐related proteins using immunostaining analysis. Analysis of 3D spheroids derived from mtMSC^MG63^, MSC, or MSC^Lipo^ cells revealed 1.5–5 fold increases in the expression of ALP, COL I, OSX, and activating transcription factor 4 in mtMSC^MG63^ compared with the control groups (MSC and MSC^Lipo^) (Figures 5B and S14, Supporting Information). We next examined cellular morphology, the degree of calcification, and the expression of the ECM to obtain further evidence of osteogenesis (Figure 5C).

Hematoxylin and eosin (H&E) staining, which distinguishes between cell nuclei and cytoplasm, revealed no abnormalities in any of the three 3D spheroid populations. This suggests that MSC^Lipo^ and mtMSC^MG63^ cells were healthy and did not experience adverse effects such as cell stress or apoptosis, which may have been induced by activation of the immune response or other complications. When 3D spheroids were stained using the Von Kossa staining method to detect calcium salts, the levels of black precipitates representing calcium salts were significantly higher in mtMSC^MG63^ samples than those in control samples, indicating advanced osteogenic differentiation. Similarly, Alizarin Red S staining detected abundant and higher levels of calcium ions in mtMSC^MG63^ spheroids than in control spheroids. Aniline Blue staining, which quantitatively assesses COL I, revealed higher levels of COL I expression in mtMSC^MG63^ spheroids than in controls. Taken together, these data indicate that increased calcification and ECM formation occurred as a result of MG63 mitochondria transfer.

### 2.7 MG63‐Derived Mitochondria Promote Bone Regeneration in a Bone Defect Model

Our results demonstrated that the transfer of MG63‐derived mitochondria into stem cells enhances their differentiation both in vitro and within 3D spheroids. Furthermore, these changes were dependent on the presence of mitochondria and were not caused by FC itself. We next wished to evaluate whether MG63‐derived mitochondria can promote tissue regeneration in vivo, using a rat model with an artificially created femoral defect.

The murine model shares ≈85% similarity with humans; however, owing to differences and the fact that it captures only a single aspect, it cannot fully represent the complexity of human biology. Additionally, there are limitations because the response can vary depending on the different strains of rodents. However, it is convenient to handle, offers good reproducibility, and is cost‐effective, which is why many studies use the murine model. Owing to physical and economic constraints, evaluating larger animals can be challenging, so it is necessary to first verify the efficacy in small animals and then proceed with a stepwise validation in larger animals. Therefore, we created a rodent model, a small animal model, to evaluate the relevant aspects.

**Figure 6:**
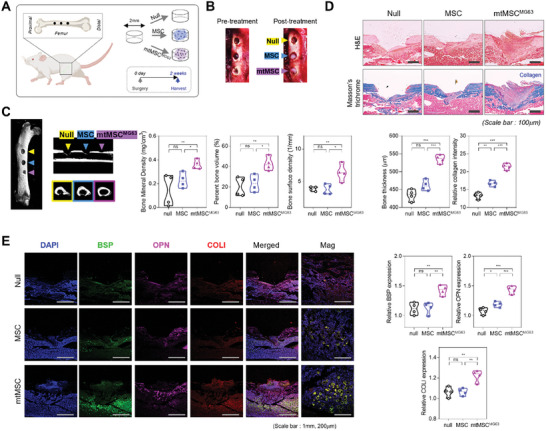
Evaluation of in vivo bone regeneration and osteogenic differentiation protein expression after MG63‐derived mitochondria transfer into a bone defect. A) Schematic diagram of the in vivo experimental protocol for transplantation of each spheroid into a bone defect rat model. B) Images of the surgical procedure for transplanting MSCs and mtMSC^MG63^ spheroid into the rat femur defect site. C) Micro‐CT images and quantitative analysis of the femur after transplantation of MSC and mtMSC^MG63^ spheroids (*n* = 4). D) Histological staining analysis of the femur after transplantation of MSC and mtMSC^MG63^ spheroids (*n* = 4). Scale bar = 100 µm. E) Immunostaining analysis of osteogenic differentiation protein expression in the femur after transplantation of MSCs and mtMSC^MG63^ spheroids (*n* = 4). Scale bar = 1 mm and 200 µm. All data are presented as average ± SD. Statistical analysis was performed using the unpaired Student *t*‐test; *p < 0.05, **p < 0.01, ***p < 0.001, and ****p < 0.0001.

An artificial 2‐mm defect was created in the femur of rats, and three experimental groups were established: a control group with an untreated defect (null), a group with MSCs transplanted alone (MSC), and a group with MSCs transplanted alongside MG63‐derived mitochondria (mtMSC^MG63^) (**Figure** **6**A). Figure 6B provides an overview of the surgical procedure and the grouping of the experimental rats. The figure highlights the systematic approach used to evaluate the regenerative capacity of MSCs under different conditions, setting a clear basis for the subsequent analysis of repair outcomes.

Micro‐computed tomography (micro‐CT) analysis showed that bone regeneration was significantly higher in the mtMSC^MG63^ group than in the null and MSC groups (Figure 6C). Metrics such as bone mineral density, bone volume, and bone surface density were significantly higher in this group than in the MSC‐only and control groups. This indicates that MG63‐derived mitochondria increased bone formation and repair more effectively than MSCs alone. Histological examination using H&E and Masson's trichrome staining revealed that the mtMSC^MG63^ group exhibited extensive tissue repair and collagen formation, in contrast to the minimal repair in the null group and partial repair in the MSC group (Figure 6D). The extensive collagen‐rich fibrous matrix in the mtMSC^MG63^ group supports the notion that MG63‐derived mitochondria significantly increase the osteogenic potential of MSCs, leading to superior tissue restoration. Immunofluorescence staining revealed strong expression of key bone differentiation proteins such as bone sialoprotein, COL I, and OPN in the mtMSC^MG63^ group (Figure 6E), suggesting that MG63‐derived mitochondria not only promote tissue repair but also facilitate the osteogenic differentiation of MSCs. The results support the conclusion that MG63‐derived mitochondria significantly contribute to effective bone regeneration and suggest their potential for clinical applications in bone tissue engineering.

These findings highlight the significant advantage of using MG63‐derived mitochondria in enhancing bone regeneration. The near‐complete recovery observed in the mtMSC^MG63^ group, compared with the partial or minimal recovery in the null and MSC groups, suggests that MG63 mitochondria play a crucial role in facilitating bone repair and promoting osteogenic differentiation. The enhanced collagen and ECM formation, along with the increased expression of bone differentiation markers, further supports the efficacy of MG63‐derived mitochondria in driving osteogenesis.

Additionally, it has been discovered that osteoblasts naturally interact with various cells, exchanging mitochondria to regulate the balance of bone tissue. Osteoblasts naturally transfer mitochondria to myeloid cells, and inhibiting mitochondrial transfer in osteoblasts leads to the differentiation of myeloid cells into osteoclasts, promoting bone loss and inducing osteoporosis.^[^ ^35^ ^]^ Furthermore, osteoblasts transfer mitochondria to epithelial cells to promote angiogenesis.^[^ ^36^ ^]^ Conversely, in osteoporotic conditions, stressed macrophages transfer mitochondria to MSCs, disrupting the balance of bone tissue.^[^ ^37^ ^]^

In line with these studies, we argue that osteoblast‐derived mitochondria play a critical role in bone formation. We emphasize that osteoblast mitochondria determine the lineage of bone marrow‐derived MSCs toward osteoblasts. However, the key difference between our findings and previous studies is that we demonstrated mitochondrial transfer not through natural intercellular transfer but via mitochondria encapsulated in cationic liposomes. This is significant because it allows us to elucidate the action and mechanisms of mitochondria and demonstrate the potential of using this action as a therapeutic approach in animal models. Our invention of artificial mitochondrial transfer technology will lead to advances in the field of Mitotherapy (Mitochondria transplantation therapy). Additionally, by revealing the potential of mitochondria to regulate stem cell lineage, we provided new insights into potential approaches for stem cell therapies.

## 3 Conclusion

This study effectively demonstrated that transplanting mitochondria from MG63 osteoblast precursor cells into MSCs significantly enhances osteogenic differentiation and tissue regeneration. MG63‐derived mitochondria, characterized by higher metabolic activity, positively influenced the energy metabolism and gene expression of recipient MSCs, promoting their differentiation into osteocytes. Key osteogenic pathways, including BMP and Wnt/β‐catenin signaling, were activated, leading to the upregulation of essential bone formation markers. Importantly, mitochondrial transfer did not induce oncogene expression, demonstrating the safety of this approach. In vivo experiments using a rat femoral defect model showed that MSCs with MG63‐derived mitochondria facilitated near‐complete bone regeneration and improved bone density. These findings underscore the potential of mitochondrial transplantation as an effective strategy for optimizing stem cell‐based bone tissue engineering. Furthermore, mitochondrial transfer not only enhanced the bioenergetic capacity of the recipient cells but also induced key metabolic and epigenetic changes, driving specific cellular fates.

We have designed our methodology and materials with large‐scale applications in mind. In particular, the type of lipids used are clinically tested products, and the synthesis method follows traditional techniques that do not require specialized technologies, ensuring the quality and stability of the liposomes. Therefore, large‐scale production can be smoothly achieved through existing manufacturing processes. Additionally, based on previous research, the mitochondrial isolation and encapsulation techniques have been standardized, ensuring batch reproducibility and minimizing barriers to clinical application by achieving consistent results. Furthermore, in both the previous and current studies, the mitochondrial delivery system was applied to the local regions of rodent disease models. As a result, no immune activation or side effects were observed at the treated local sites. However, future studies should assess potential side effects, such as immune responses, when the administration route is altered.

This study suggests that mitochondria can regulate MSC differentiation, with the direction of differentiation being determined by the cell of mitochondrial origin. Since it has been shown that mitochondria can regulate cell behavior, it is necessary to investigate whether mitochondria from specific diseases can have therapeutic effects. Therefore, we plan to apply this to disease models, delivering and transplanting appropriate mitochondria to study their efficacy as potential therapeutics.

## 4 Experimental Section

### Materials

Materials used for fusogenic liposome and mitochondrial isolation were described previously.^[^ ^10^ ^]^ Dulbecco's modified Eagle's medium, high glucose, fetal bovine serum, and Dulbecco's phosphate‐buffered saline were purchased from HyClone (Logan, Canada). Antibiotic‐antimycotic solution and trypsin‐EDTA were purchased from Gibco (Massachusetts, USA). Mitotracker Green FM, Mitotracker Deep Red FM, TMRE and JC‐1 dye, and Fluo‐4 AM were purchased from Invitrogen (CA, USA).

### Cell Culture and MT Isolation from Donor Cells

Human MSCs were obtained from Lonza (Basel, Switzerland), and human osteosarcoma MG63 and rat myoblast L6 cells were obtained from ATCC (Virginia, USA). All cells were cultured in Dulbecco's modified Eagle's medium, high glucose (SH30243.01, HyClone) supplemented with 10% fetal bovine serum and 1% antibiotic‐antimycotic solution in 5% CO_2_ at 37 °C. The culture medium was replaced every 2–3 days, and experiments were mainly conducted with cells at passages 7–9. MTs were isolated from each cell through differential centrifugation using the MT Isolation Kit (89874, Thermo Fisher Scientific) as described previously.^[^ ^10^ ^]^

### RNA Isolation

Total RNA was isolated using Trizol reagent according to the manufacturer's instructions (Invitrogen), and its quality was assessed using the TapeStation4000 System (Agilent Technologies, Amstelveen, The Netherlands). Quantification of RNA was conducted using the ND‐2000 Spectrophotometer (Thermo Fisher Scientific, USA).

### Library Preparation and Sequencing

Libraries were prepared from total RNA using the CORALL RNA‐Seq V2 Library Prep Kit (LEXOGEN, Austria). mRNA was isolated with the Poly(A) RNA Selection Kit (LEXOGEN, Austria), and the resulting mRNAs were used for cDNA synthesis and shearing according to the manufacturer's instructions. Indexing was performed with Illumina indexes 1–12, and the enrichment step was conducted using PCR. The libraries were then checked using the TapeStation HS D1000 Screen Tape (Agilent Technologies, Amstelveen, The Netherlands) to assess the mean fragment size. Quantification was conducted with the library quantification kit on a StepOne Real‐Time PCR System (Life Technologies, USA). Finally, high‐throughput sequencing was performed as paired‐end 100 sequencing using the NovaSeq 6000 (Illumina, USA).

### RNA‐Seq Data Pre‐Processing and Analysis

Fastq files, which contain raw sequencing data from RNA‐sequencing, underwent raw read quality control using FastQC (v0.11.9)^[^ ^38^ ^]^ and MultiQC (v1.18).^[^ ^39^ ^]^ The files were processed using Fastp (v0.23.1)^[^ ^40^ ^]^ to remove low‐quality reads and adapters. Paired‐end reads were then mapped to the human reference genome (GRCh38) using STAR (v2.7.10.b),^[^ ^41^ ^]^ and the number of reads that were mapped at the gene level was calculated using Salmon (v1.10.0).^[^ ^42^ ^]^ Raw count data were normalized using the DESeq2 R package (v1.42.1).^[^ ^43^ ^]^

DE analysis was performed using the DESeq2 R package. Significant DEGs were selected with the criteria *p* <  0.05 and absolute log2 fold change (|Log2FC|) > 1. GO analysis was performed using g:Profilier^[^ ^44^ ^]^ to facilitate elucidation of the molecular function, the biological process, and the cellular component of identified DEGs. Gene set enrichment analysis was also conducted using the ClusterProfiler R package (v4.10.1)^[^ ^45^ ^]^ with normalized expression data to identify significant pathways in all genes. Specifically, to observe the interactions between key genes, a protein‐PPI network was constructed using Cytoscape (v3.10.2)^[^ ^46^ ^]^ based on the STRING database (v12.0).^[^ ^47^ ^]^ Based on the genes in a PPI network, hub genes were screened using cytoHubba^[^ ^48^ ^]^ and ranked using the maximal clique centrality method. Statistical analysis and plotting were conducted using R (v4.1.3),^[^ ^49^ ^]^ supplemented with the ggplot2 R package (v3.5.1).^[^ ^50^ ^]^

### MT and FMC Characterization

To observe the morphology of MTs in each cell, cells were harvested and fixed with 4% paraformaldehyde (PFA) for 0.5 h. After fixation, the cells were embedded in resin. The blocks were then sectioned, stained with uranyl acetate, washed, and observed using TEM (Hitachi, Tokyo, Japan). For fluorescence imaging, MTs were labeled with either Mitotracker Green FM (M7514, Invitrogen) or Deep Red (M22426, Invitrogen) and subsequently visualized using CLSM (Olympus FV3000, Tokyo, Japan). For the Seahorse assay (103015‐100, Agilent), MSCs were seeded at 4.0 × 10^3^ and MG63 cells at 6.0 × 10^3^ in Seahorse cell culture plates (103774‐100, Agilent), and the protocol was performed according to the manufacturer's instructions.

Fusogenic liposomes were synthesized as described previously.^[^ ^10^ ^]^ FMCs were formed by mixing isolated MT and FCs at a mass ratio of 1:1. The size and ZP of each MT and FMC were measured using dynamic light scattering (Zetasizer Nano ZS, Malvern Panalytical, Malvern, UK). To measure MT encapsulation efficiency, the isolated MT and FC were detected using violet side scatter in a CytoFLEX flow cytometer (Beckman Coulter, CA, USA). Each isolated MT was stained with Mitotracker Deep Red (M22426, Invitrogen), and non‐fluorescent FC was synthesized by removing Liss Rhod PE from the FC. Subsequently, isolated MTs and FCs were zoned using the non‐staining groups, and the areas where MTs and FCs fluoresced simultaneously were characterized as FMC, and the MT encapsulation efficiency of FMC was quantified.

### MT Uptake and Evaluation of Delivered MT Effects

To perform imaging of cellular membrane fusion to fusogenic liposomes for analysis of MT intracellular delivery, 8.0 × 10^3^ MSCs were seeded in a 96‐well cell culture black plate (33396, SPL), and 0.16 µg of FMC labeled with Mitotracker Green FM was delivered to the cells. The time‐lapse observation was performed using CLSM for 0.5 h.

To observe the presence of delivered mitochondria in cells, MSCs were seeded at 2.0 × 10^5^ in 6‐well cell culture plates (140675, Thermo Fisher Scientific), and each FMC was delivered at 4 µg for 0.5 h. The cells were then harvested and observed using TEM, and the sampling process was as described above.

To quantify MT delivery using the xenograft method, L6 cells were seeded at 2.0 × 10^5^ in 6‐well cell culture plate (140675, Thermo Fisher Scientific), and 4 µg of FMC of MSC and MG63 was delivered to L6 cells for 0.5 h. After genomic DNA preparation (106‐101, GeneAll), genomic DNA was analyzed using PCR and visualized using a gel documentation imaging system (BR170‐8265; Bio‐Rad Laboratories, Korea). The primers were used as described previously.^[^ ^10^ ^]^

### TMRE and JC‐1 Staining Analysis

For TMRE (T669, Invitrogen) and JC‐1 (T3168, Invitrogen) staining, MSCs were seeded in 96‐well black plates (33396, SPL) at 8.0 × 10^3^. Subsequently, 0.16 µg of each FMC was delivered for 0.5 h. After 4 h, cells were stained with TMRE and JC‐1 and observed using CLSM. In this experiment, non‐fluorescent FC was used, TMRE was stained at 1 µm for 0.5 h, and JC‐1 was stained at 1 µg mL^−1^ for 0.5 h. For quantitative data, the same experiment was performed on 96‐well black plates (30296, SPL), and fluorescence was measured using a microplate reader (SpectraMax‐ID5, Molecule Devices, CA, USA).

### Fluo‐4 Staining Analysis

For Fluo‐4 (F14201, Invitrogen) staining, MSCs were seeded in 96‐well black plates (33396, SPL) at 8.0 × 10^3^, and then, 0.16 µg of each FMC was delivered for 0.5 h. During the 0.5 h delivery of each FMC, time‐lapse CLSM images were acquired every 3 min. Afterward, the intensity of fluo‐4 was quantified using CellSens software (Olympus, Japan).

### Analysis of mRNA and Protein Levels of mtMSCs in 2D Culture

MSCs were seeded in a 6‐well cell culture plate (140675, Thermo Fisher Scientific) at 2.0 × 10^5^, and each FMC was delivered at 4 µg for 0.5 h. Cells were harvested 24 h later, and qRT‐PCR was performed to analyze the mRNA levels of each marker. Total RNA was extracted from each group using an RNA prep kit (K‐3140, Bioneer, Korea), and cDNA was synthesized using M‐MLV Reverse Transcriptase (28025013, Invitrogen). Real‐time PCR was performed using SYBR Green master mix (Takara, Japan) and the primers listed in Table S1 (Supporting Information) under appropriate conditions for each primer set.

For western blotting analysis, cells were lysed with radioimmunoprecipitation buffer and quantified using bicinchoninic acid assay (23225, Thermo Fisher Scientific). Protein lysate (30 µg) was separated on an 8–12% sodium dodecyl sulfate–polyacrylamide gel electrophoresis gel and transferred onto a polyvinylidene fluoride membrane. After blocking, membranes were incubated with primary antibodies against the target protein, followed by horseradish peroxidase‐conjugated secondary antibodies corresponding to the species in which the primary antibody was raised, and protein signals were detected using a gel documentation imaging system (BR170‐8265; Bio‐Rad Laboratories, Korea).

### Spheroid Culture and Staining

Cells were treated with the appropriate liposome (FC or FMCMG63) at a concentration of 4 µg for 0.5 h, and then the spheroid culture was performed for seven days with 5.0 × 10^5^ cells in each group. Western blotting was performed as described above to evaluate osteogenic differentiation. For staining spheroid sections, each spheroid was fixed with 4% PFA, dehydrated with 20% sucrose, embedded in OCT compound, frozen, and then cryosectioned (Leica, Wetzlar, Germany).

For immunofluorescence, each section was permeabilized with 0.1% Triton X‐100, blocked with 1% bovine serum albumin, and incubated with primary antibodies against the target proteins. Following incubation with the secondary antibody, DNA was stained with 4′,6‐diamidino‐2‐phenylindole. The sections were mounted onto glass slides with Canada balsam (C0249, SAMCHUN chemical), observed with CLSM, and quantified with CellSens software.

H&E, Alizarin Red S, Von Kossa, and aniline blue staining were all performed according to the manufacturer's protocol after removing the OCT compound from each section. Stained samples were then visualized with an optical microscope (EVOS XL Core, AMEX1000, Invitrogen, USA).

### In Vivo Experiments

The animal study was approved by the Institutional Animal Care and Use Committee (IACUC) of CHA University, Korea (Approval No. IACUC230175). Eight‐week‐old SD rats were purchased from Raonbio (Yongin‐si, Korea).

To create the femoral defect, a hole 2 mm long, 2 mm wide, and 2 mm deep was drilled in the femur of an isoflurane‐anesthetized rat. No cells (Null), MSC spheroids, or mtMSC^MG63^ spheroids were transplanted into the hole. Two weeks after the surgery, the rats were sacrificed, and the femurs were harvested. The harvested femurs were fixed with 4% PFA and subjected to micro‐CT imaging and histological analysis. Micro‐CT imaging and bone density analysis were performed using a micro‐CT scanner (skyscan 1173; Bruker, Massachusetts, USA). For histological analysis, the femurs were embedded in paraffin blocks. After paraffin sectioning and deparaffinization, immunofluorescence, H&E, and Masson's trichrome staining were performed as described above.

### Statistical Analysis

In RNA‐seq data preprocessing and analysis, raw sequencing data in Fastq files underwent quality control using FastQC (v0.11.9)^[^ ^38^ ^]^ and MultiQC (v1.18).^[^ ^39^ ^]^ Low‐quality reads and adapter sequences were removed using Fastp (v0.23.1).^[^ ^40^ ^]^ The cleaned paired‐end reads were then mapped to the human reference genome (GRCh38) using STAR (v2.7.10b),^[^ ^41^ ^]^ and gene‐level read counts were quantified using Salmon (v1.10.0).^[^ ^42^ ^]^

Normalization of raw count data was performed using the DESeq2 R package (v1.42.1).^[^ ^43^ ^]^ DE analysis was also conducted using DESeq2, with significant DEGs identified based on the criteria of *p* <  0.05 and absolute log2 fold change (|Log2FC|) > 1. DEG *p* values were calculated using the Wald test and adjusted for multiple testing using the Benjamini‐Hochberg (BH) method. However, as fewer than five DEGs were identified between MSC and mtMSCMG63 under the adjusted *p* value threshold (*p* <  0.05), the selection of DEGs was based on raw *p* values.

GO analysis was conducted using g:Profiler^[^ ^44^ ^]^ to elucidate the molecular functions, biological processes, and cellular components associated with the identified DEGs. GO analysis *p* values were calculated using Fisher's exact test and adjusted with the False Discovery Rate (FDR) method. GSEA was performed using the ClusterProfiler R package (v4.10.1)^[^ ^45^ ^]^ with normalized expression data to identify significant pathways across all genes. Pathway significance *p* values were calculated via permutation tests and adjusted using the FDR method.

To investigate interactions among key genes, a protein–protein interaction (PPI) network was constructed using Cytoscape (v3.10.2).^[^ ^46^ ^]^ based on data from the STRING database (v12.0).^[^ ^47^ ^]^

All statistical analyses were performed using R 4.1.3 software and GraphPad Prism 8.0 software. Data are presented as the mean ± standard deviation. A Student's *t*‐test was conducted to assess significant differences between the two groups, assuming normal distribution and equal standard deviations. *P*‐values were considered statistically significant as follows: *p <  0.05, **p <  0.01, and ***p <  0.001. Sample size (*n*) for each analysis and specific statistical tests are described in the corresponding figure legends. All boxplots represent the median (center), 25th and 75th percentiles (boundaries of the box), 95% confidence interval (error bars). All plots were conducted with the ggplot2 R package (v3.5.1).^[^ ^50^ ^]^

## Conflict of Interest

The authors declare no conflict of interest.

## Author Contributions

H.R.K.: Formal analysis, investigation, methodology, resources, visualization, validation, writing—original draft preparation, writing—review and editing; S.W.: Data curation, software, formal analysis, visualization, writing—original draft preparation; H.B.C.: Investigation, visualization; S.L.: Investigation, visualization; C.W.C.: Investigation; S.Y.: Resources; G.S.: Resources; S.K.: Validation; J.‐I.P.: Validation; S.H.: Project administration, supervision, writing—original draft preparation; H.J.K.: Formal analysis, methodology, project administration, visualization, writing—original draft preparation, writing—review and editing; K.‐H.P.: Conceptualization, funding acquisition, project administration, supervision, writing—original draft preparation

## Supporting information



Supporting Information

## Data Availability

The data that support the findings of this study are available from the corresponding author upon reasonable request. The bulk RNA‐seq data have been deposited to the Gene Expression Omnibus database with the accession code GSE279815. The raw data are provided with this paper.
