# Supplementary material for: Osteoblast‐Derived Mitochondria Formulated with Cationic Liposome Guide Mesenchymal Stem Cells into Osteogenic Differentiation
Source: Adv Sci (Weinh). 2025 Jan 31;12(12):2412621. doi: 10.1002/advs.202412621 (PMC11948037; doi:10.1002/advs.202412621)
Supplement: Supplementary file 1 — Supporting Information [file ADVS-12-2412621-s001.docx]

Supporting information

**Osteoblast-derived mitochondria formulated with cationic liposome guide mesenchymal stem cells into osteogenesis**

**Hye-Ryoung Kim^1†^, Seonjeong Woo^2†^, Hui Bang Cho^1^, Sujeong Lee^1^, Chae Won Cho^1^, Ji-In Park^1^, Seulki Youn^1^, Gyuwon So^1^, Sumin Kang^1^, Sohyun Hwang^2, 3, *^, Hye Jin Kim^1, *^, and Keun-Hong Park^1, *^**

^1^ School of Bioconvergence, CHA University, 6F, CHA Biocomplex, Sampyeong-Dong, Bundang-gu, Seongnam-si, 13488, Republic of Korea.

^2^ Department of Biomedical Science, CHA University, Seongnam, Republic of Korea.

^3^ Department of Pathology, CHA Bundang Medical Center, CHA University School of Medicine, Seongnam, Republic of Korea.

^†^these authors contributed equally to this work.

**[*] co-corresponding authors**

**Sohyun Hwang:** [blissfulwin@cha.ac.kr](mailto:blissfulwin@cha.ac.kr)

**Hye Jin Kim:** [khye4680@naver.com](mailto:khye4680@naver.com)

**Keun-Hong Park:** [pkh0410@cha.ac.kr](mailto:pkh0410@cha.ac.kr)

**Materials and Method**

**1. Materials**
The CellVia cell viability WST-1 assay kit was purchased from Abfrontier (Seoul, Korea). The Seahorse glycolytic rate assay kit was purchased from Agilent (CA, USA) and the BMP2 ELISA kit was purchased from RnD systems (MN, USA).

**2. Cytotoxicity and confirmation of fusogenic capsule (FC)**
To evaluate the cytotoxicity of FC to MSCs, a WST-1 assay was performed. Briefly, 1.0 x 10^4^ MSCs were seeded in 96-well cell culture plates (30096, SPL), and FC was administered in a time-dependent and dose-dependent manner. Subsequently, WST-1 reagent was added and the absorbance at 450 nm was measured 2 h later using a microplate reader.
To confirm the efficiency of MT delivery through fusogenic mito-capsule (FMC), xenograft experiments were performed. MSCs were seeded at a density of 2.0 x 10^5^ in 6-well cell culture plates (140675, Thermo Fisher Scientific), and 4 μg of MT isolated from L6 was delivered to MSCs in the form of naked MT and FMC. Each group was treated proportionally to the amount of MT, and cells were harvested after 1 h. Each mtDNA was detected by PCR and visualized using a gel documentation imaging system (BR170-8265; Bio-Rad Laboratories, Korea).

**3. Metabolic analysis of MSC after MT delivery**
For the seahorse assay (103015-100, Agilent) and glycolytic rate assay (103344-100, Agilent), MSCs were seeded at a density of 4.0 x 10^3^ in Seahorse cell culture plates (103774-100, Agilent) and then treated with FC or FMC^MG63^ for 0.5 h. After 24 h, the glycolytic rate assay was performed according to the manufacturer's instructions.

**4. BMP2 ELISA analysis**

MSCs were seeded in 6-well cell culture plates (140675, Thermo Fisher Scientific) at 2.0 x 10^5^, and each FMC was delivered at 4 μg for 0.5 h. Cell media were harvested at each time point and a BMP2 ELISA was performed according to the manufacturer's instructions.

**5. Western blotting analysis of b-catenin inhibition and activation**

In the β-catenin inhibition experiment, IWP2 (72122, STEMCELL) was pretreated at 10 μM for 48 hours before delivering FMC^MG63^ (4 μg) for 0.5 hours. Cells were harvested 1 hour after delivery. In the β-catenin activation experiment, Forskolin (F6886, Sigma-Aldrich) was pretreated at 50 μM for 0.5 hours, followed by FMC^MG63^ delivery (4 μg) for 0.5 hours. Cells were harvested immediately after delivery and 24 hours later.

Cells were lysed in RIPA buffer for Western blotting analysis, and protein concentration was quantified using a BCA assay kit (23225, Thermo Fisher Scientific). Protein lysates (35 μg) were separated using 10% SDS-PAGE gels and transferred onto PVDF membranes. After blocking, membranes were incubated with primary antibodies specific to the target proteins, followed by HRP-conjugated secondary antibodies. Protein signals were detected using a gel documentation imaging system (BR170-8265; Bio-Rad Laboratories, Korea).

**Table S1. Sequences of qRT-PCR primers used.**

| Gene | Sequence (5’→3’) | |
| --- | --- | --- |
| OCN | Forward | CCAGGCGCTACCTGTATCAA |
|  | Reverse | AGGGGAAGAGGAAAGAAGGG |
| OPN | Forward | CATCTCAGAAGCAGAATCTC |
|  | Reverse | CCATAAACCACACTATCACC |
| ALP | Forward | TAACATCAGGGACATTGACG |
|  | Reverse | TGCTTGTATCTCGGTTTGAA |
| COLI | Forward | AGAACATCACCTACCACTGC |
|  | Reverse | ATGTCCAAAGGTGCAATATC |
| COLII | Forward | ATGACAATCTGGCTCCCAAC |
|  | Reverse | GAACCTGCTATTGCCCTC |
| ATF4 | Forward | CTGACCACGTTGGATGACAC |
|  | Reverse | GGGCTCATACAGATGCCTCT |
| RUNX2 | Forward | CCGCACGACAACCGCACCAT |
|  | Reverse | CGCTCCGGCCCACAAATCTC |
| OSX | Forward | TCCTCCTGCGACTGCCCTAA |
|  | Reverse | TGCGAAGCCTTGCCATACA |
| SOX9 | Forward | GGTGCTCAAGGGCTACGACT |
|  | Reverse | GGGTGGTCTTTCTTGTGCTG |
| COMP | Forward | CAGGACGACTTTGATGCAGA |
|  | Reverse | AAGCTGGAGCTGTCCTGGTA |
| C/EBPα | Forward | CGGTGGACAAGAACAGCAAC |
|  | Reverse | CGGAATCTCCTAGTCCTGGC |
| SREBP1 | Forward | ACAGTGACTTCCCTGGCCTAT |
|  | Reverse | CATGGACGGGTACATCTTCAA |
| Adiponectin | Forward | AGCCTCCTTCTCCTGGGTCC |
|  | Reverse | GTTGCCTCTAGCCTGGTGGG |
| NBL1 | Forward | TGTTCCCAGATAAGAGTGCCT |
|  | Reverse | GCAGGAGTCACAGTGAACCAG |
| PLS1 | Forward | GGTGAGGAACTAGAGGAGCTG |
|  | Reverse | TGGCTGAAGTTGCTGATGGT |
| CDC42 | Forward | CCATCGGAATATGTACCGACTG |
|  | Reverse | CTCAGCGGTCGTAATCTGTCA |
| CORO1A | Forward | CACCAACATCGTCTACCTCTG |
|  | Reverse | ACTCCTTGGAACTGAACATGG |
| TRPV4 | Forward | TCAGGGAATCACAGTTGGC |
|  | Reverse | AGCTCTTCATTGATGGATTCTT |
| TRPV5 | Forward | TTGCCTCCGCGTTCTATATCA |
|  | Reverse | GGCAAGTCCACGTCGTAGTTG |
| TRPV6 | Forward | ACTGACCTCGACTCTCTATGAC |
|  | Reverse | GTGGTGATGATAAGTTCCAGCAG |
| WNT2 | Forward | TAGTCGGGAATCTGCCTTTG |
|  | Reverse | TTCCTTTCCTTTGCATCCAC |
| WNT3 | Forward | ACGAGAACTCCCCCAACTTT |
|  | Reverse | GATGCAGTGGCATTTTTCCT |
| GAPDH | Forward | CGCTGAGTACGTCGTGGAGT |
|  | Reverse | ATGATGTTCTGGAGAGCCCC |


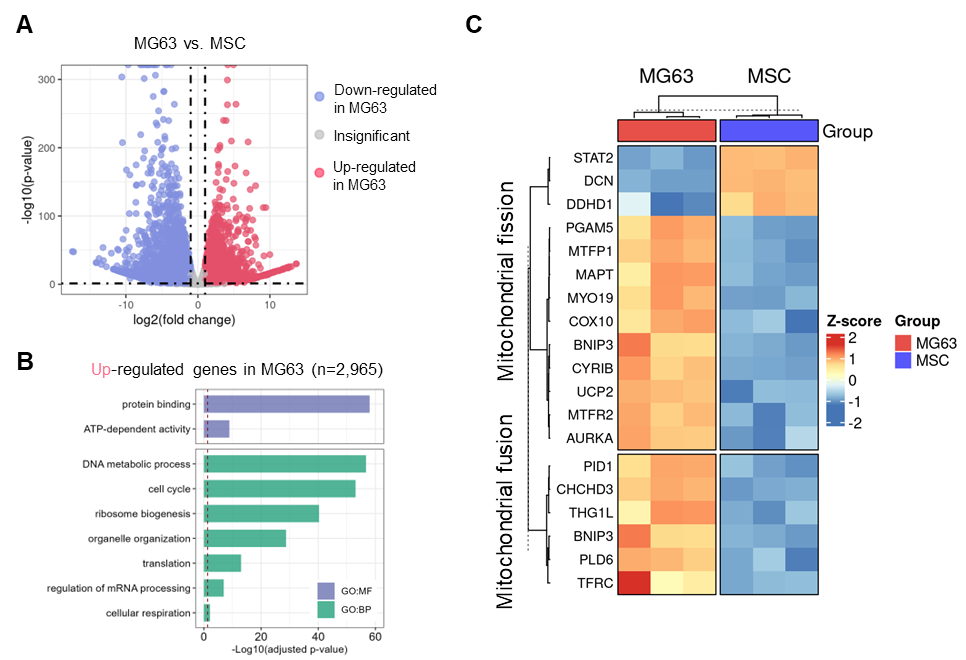


**Figure S1. Transcriptome analysis of differences between MSC and MG63.**

(A) Volcano plot and (B) GO functional analysis of upregulated DEGs showing transcriptome differences between MSC (*n* = 3) and MG63 (*n* = 3). *p* value for DEG (A) was calculated by Wald test, and *p* value for GO analysis (B) was by Fisher’s exact test. *p* value for GO plot was adjusted by FDR method. (C) Heatmaps show the expression differences of genes associated with mitochondrial dynamics between MSCs (*n* = 3, blue) and MG63 (*n* = 3, red) cells. The color of gene expression indicates z-score of each gene. A red color indicates increased expression, while a blue color indicates decreased expression.


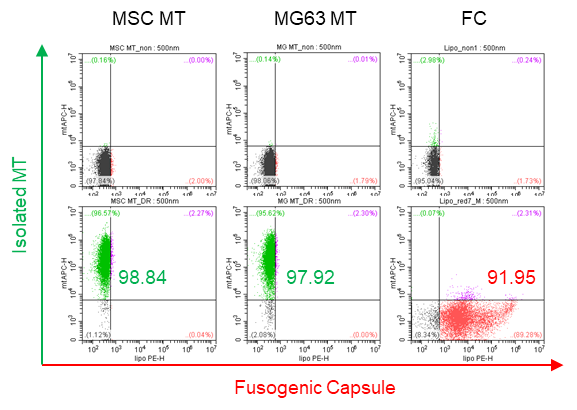


**Figure S2. Flow cytometry analysis of mitochondria and fusogenic liposomes.**


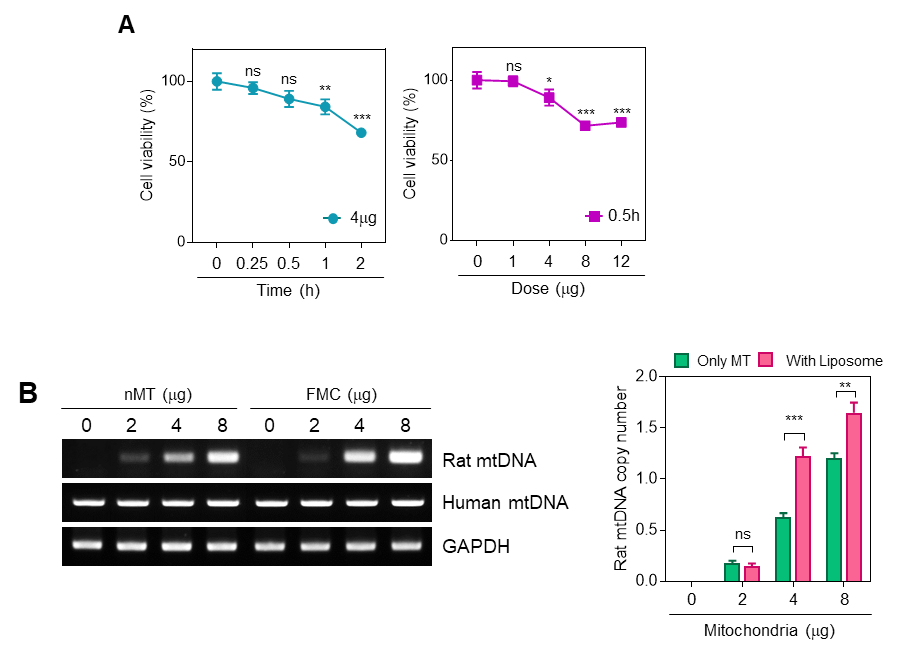


**Figure S3. Characterization and confirmation of synthesized fusogenic liposomes.**

(A) WST-1 analysis of cytotoxicity offusogenic capsule (*n* = 3). (B) PCR and quantification of rat mtDNA copy number were used to compare the efficiency of delivery of nMTs and FMCs (*n* = 3). All data are presented as average ± SD; Statistical analysis was performed using unpaired Student t-test; *P < 0.05, **P < 0.01 and ***P < 0.001).


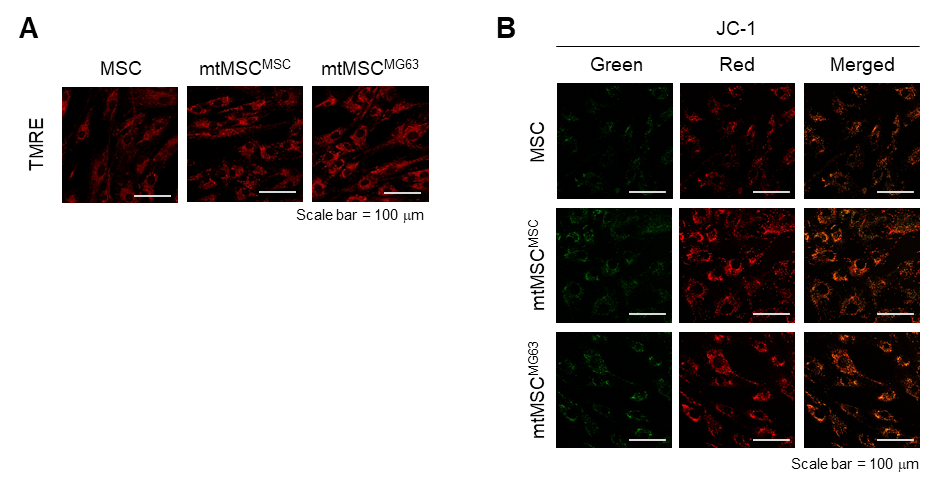


**Figure S4. Low-magnification image of recipient cells after MT delivery.**

(A) Tetramethylrhodamine ethyl ester and (B) JC-1 analysis of changes in MMP activity in recipient cells. Scale bar, 100 µm.


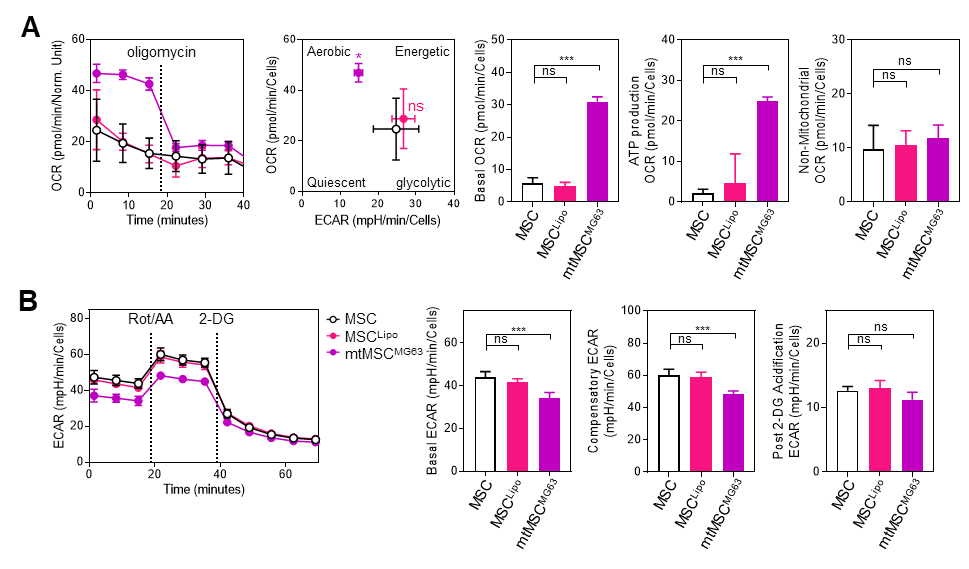


**Figure S5. Changes in cellular respiration after mitochondrial delivery.**

(A) Cell mitochondria stress assay and (B) Glycolytic rate assay after MG63 mitochondria delivery (*n* = 3). All data are presented as average ± SD; Statistical analysis was performed using unpaired Student t-test; *P < 0.05, **P < 0.01 and ***P < 0.001).


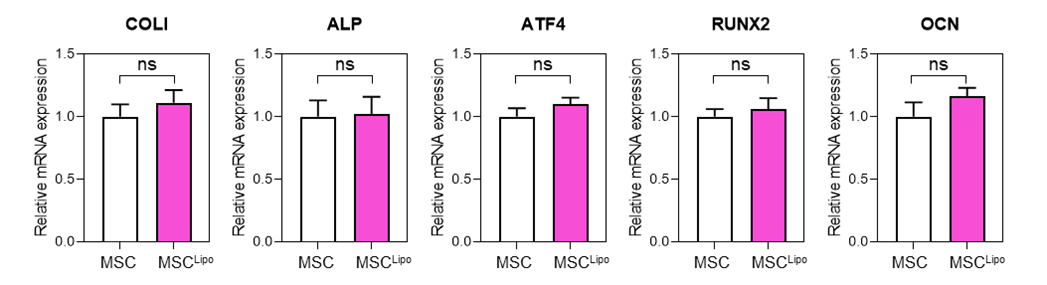


**Figure S6. qRT-PCR analysis confirming the osteogenic differentiation effect of liposomes alone.**

(*n* = 3). All data are presented as average ± SD; Statistical analysis was performed using unpaired Student t-test; *P < 0.05, **P < 0.01 and ***P < 0.001).


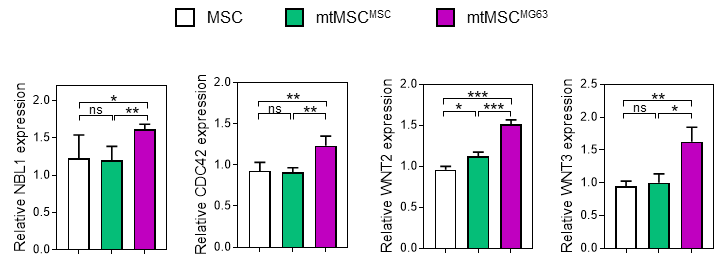


**Figure S7. qRT-PCR analysis comparing the expression levels of proteins of the BMP-Wnt/b-catenin axis.**

(*n* = 3). All data are presented as average ± SD; Statistical analysis was performed using unpaired Student t-test; *P < 0.05, **P < 0.01 and ***P < 0.001).

**Figure S8. Western blot analysis after mitochondrial transfer under β-catenin activation and inhibition.**


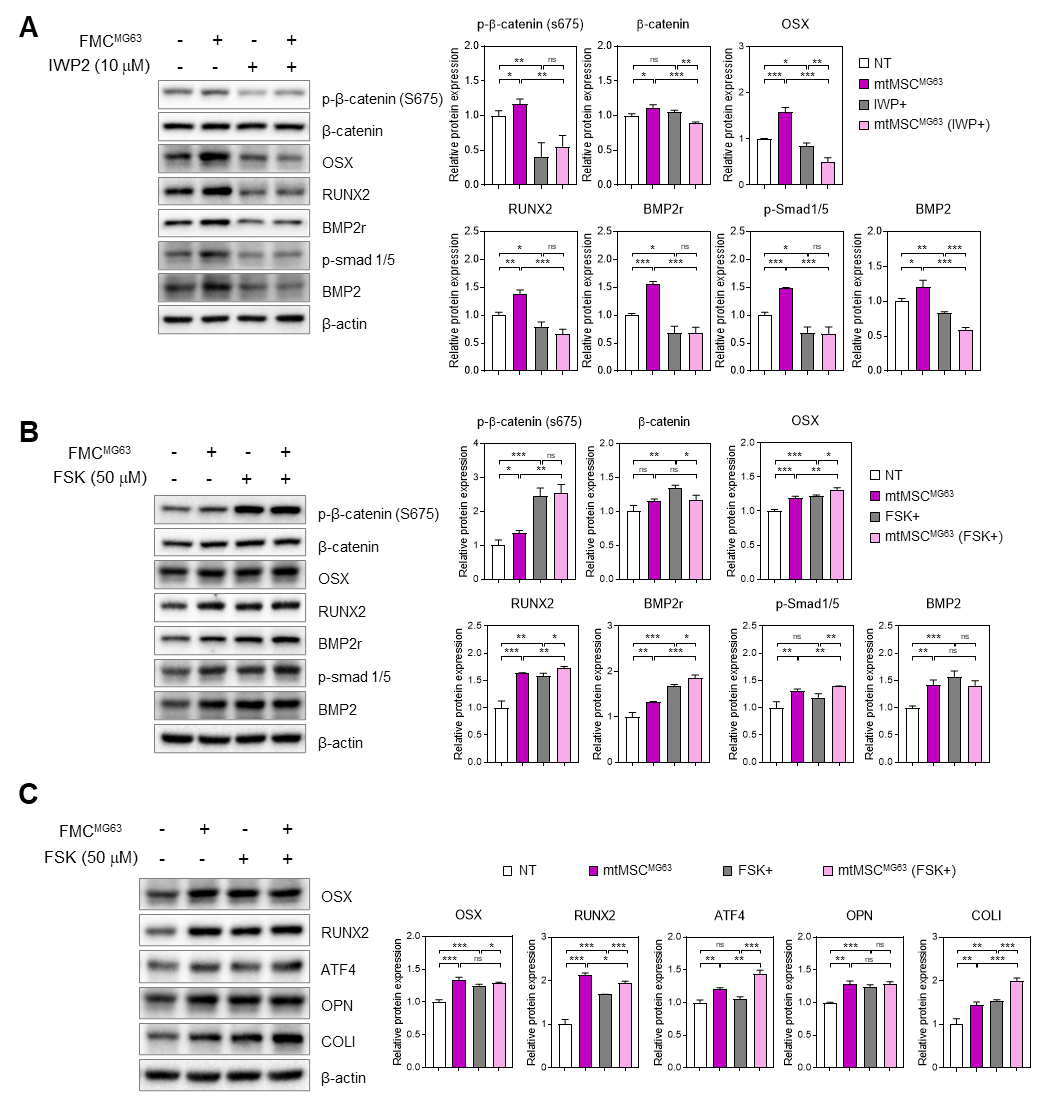


(A) Analysis of β-catenin and osteogenesis-related protein expression levels after 48 hours of IWP2 pretreatment and 0.5 hours of FMC^MG63^ treatment (n = 3). Analysis of β-catenin and osteogenesis-related protein expression levels after 0.5 hours of FSK pretreatment and 0.5 hours of FMC^MG63^ treatment (B), and 24 hours after FMC^MG63^ treatment (C) (n = 3).


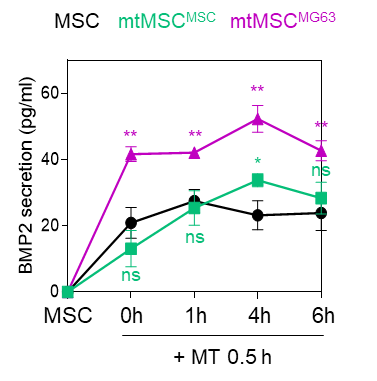


**Figure S9. ELISA analysis of BMP2 secretion over time after delivery of MSC and MG63 mitochondria.**

(*n* = 3). All data are presented as average ± SD; Statistical analysis was performed using unpaired Student t-test; *P < 0.05, **P < 0.01 and ***P < 0.001).


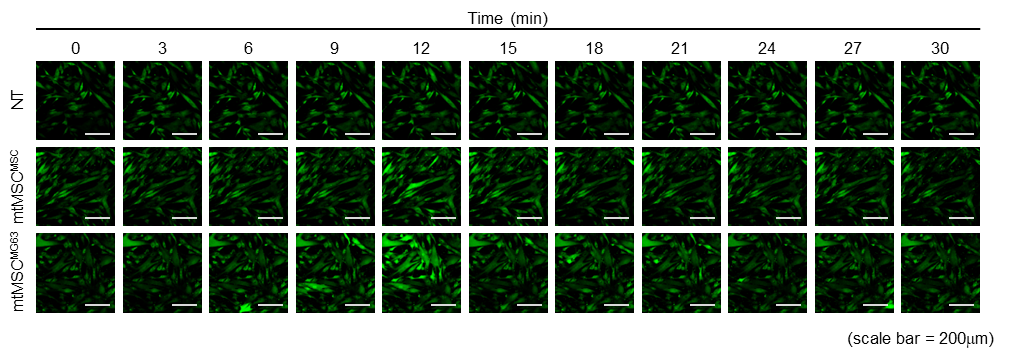


**Figure S10. Sequential confocal laser scanning microscopy images analyzing calcium influx during mitochondrial delivery. Scale bar, 200 µm.**


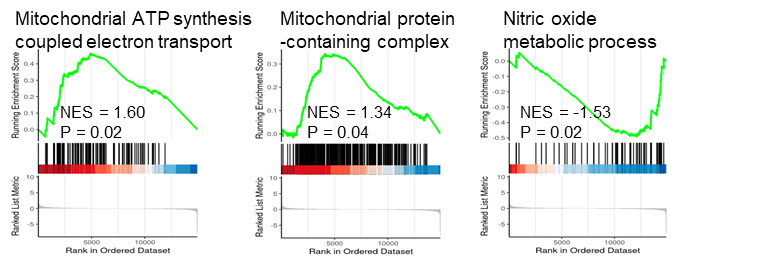


**Figure S11. Gene set enrichment analysis of osteogenic differentiation pathway-related genes in the mtMSC^MG63^ spheroid.**


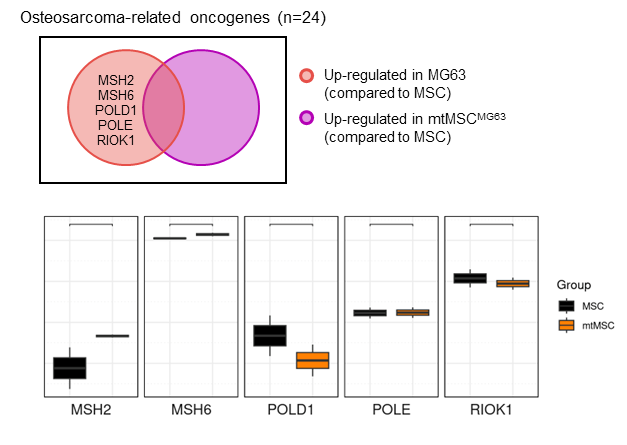


**Figure S12. Expression levels of oncogenes in mtMSC^MG63^ spheroid were compared with those in MSC spheroid using DE analysis.**

Venn diagram of five upregulated DEGs among twenty-four oncogenes associated with osteosarcoma, between mtMSC^MG63^ (n = 2, yellow) and MSC (n = 2, black). Boxplots showing the log2-scaled normalized expression of five oncogenes in mtMSC^MG63^ compared to MSC, with error bars represent the 95% confidence interval. DEGs were classified based on a *p* value <0.05 and |log2FC| >1. *p* values were calculated with the Wald test.


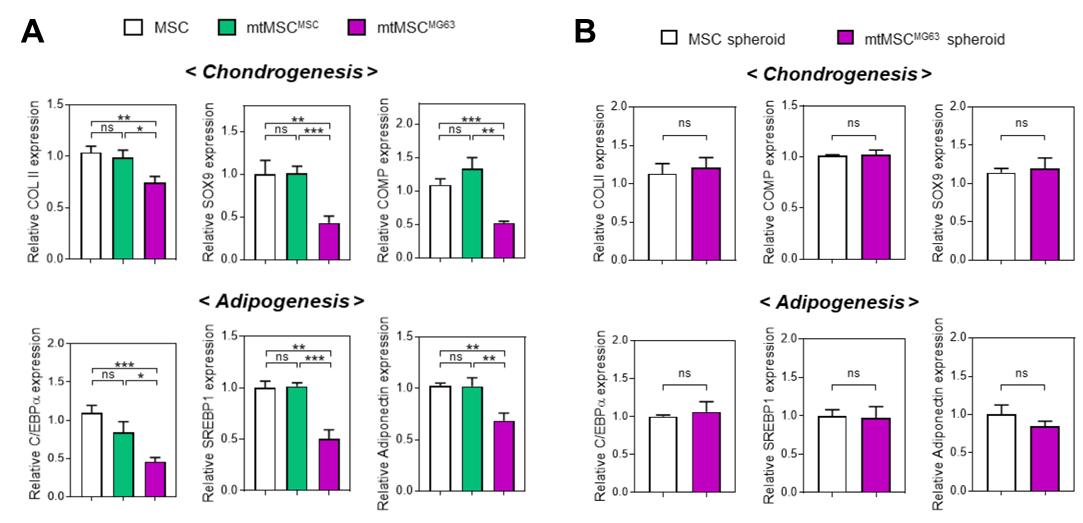


**Figure S13. qRT-PCR analysis confirming the effects of mtMSC^MSC^ and mtMSC^MG63^ on other differentiation pathways (adipogenesis and chondrogenesis), performed (A) 24 hours and (B) 7 days after each mitochondrial delivery.**

(*n* = 3). All data are presented as average ± SD; Statistical analysis was performed using unpaired Student t-test; *P < 0.05, **P < 0.01 and ***P < 0.001).


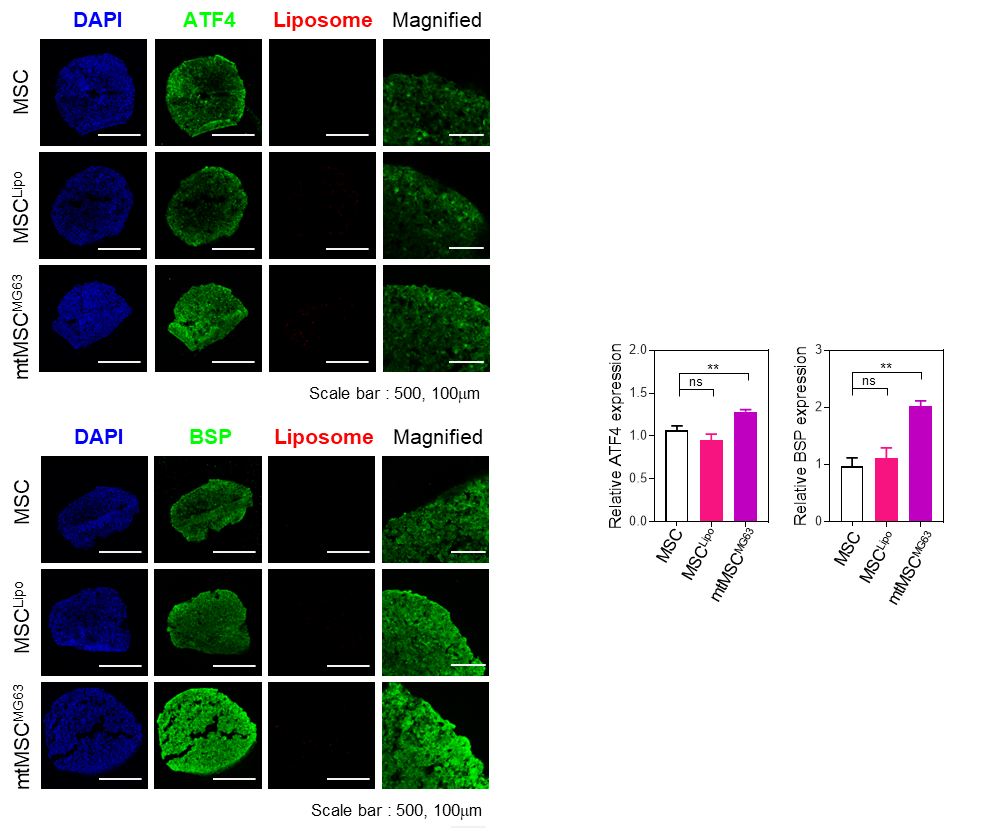


**Figure S14. Immunostaining analysis for ATF4 and BSP protein expression in spheroid sections of MSC, mtMSC^Lipo^, and mtMSC^MG63^. Scale bar, 500 µm and 100 µm.**

(*n* = 3). All data are presented as average ± SD; Statistical analysis was performed using unpaired Student t-test; *P < 0.05, **P < 0.01 and ***P < 0.001).
